# Behavioral and Molecular Responses to Exogenous Cannabinoids During Pentylenetetrazol-Induced Convulsions in Male and Female Rats

**DOI:** 10.3389/fnmol.2022.868583

**Published:** 2022-08-09

**Authors:** Antonella Zirotti Rosenberg, Maxs Méndez-Ruette, Mario Gorziglia, Benjamín Alzerreca, Javiera Cabello, Sofía Kaufmann, Lukas Rambousek, Andrés Iturriaga Jofré, Ursula Wyneken, Carlos A. Lafourcade

**Affiliations:** ^1^Escuela de Biotecnología, Facultad de Ciencias, Universidad Mayor, Santiago, Chile; ^2^Centro de investigación e innovación Biomédica (CiiB), Laboratorio de Neurociencias, Universidad de Los Andes, Santiago, Chile; ^3^Facultad de Medicina, Universidad de Los Andes, Santiago, Chile; ^4^Institute of Experimental Immunology, University of Zurich, Zurich, Switzerland; ^5^Facultad de Ciencia, Universidad de Santiago de Chile, Santiago, Chile; ^6^IMPACT, Center of Interventional Medicine for Precision and Advanced Cellular Therapy, Santiago, Chile; ^7^Department of Biological Sciences, Xi’an Jiaotong-Liverpool University (XJTLU), Suzhou, China

**Keywords:** cannabinoids, seizures, epilepsy, beta arrestin-2, hippocampus, dentate gyrus

## Abstract

Epilepsy is a disabling, chronic brain disease,affecting ~1% of the World’s population, characterized by recurrent seizures (sudden, uncontrolled brain activity), which may manifest with motor symptoms (e.g., convulsions) or non-motor symptoms. Temporal lobe epilepsies (TLE) compromising the hippocampus are the most common form of focal epilepsies. Resistance in ~1/3 of epileptic patients to the first line of treatment, i.e., antiepileptic drugs (AEDs), has been an important motivation to seek alternative treatments. Among these, the plant *Cannabis sativa* (commonly known as marihuana) or compounds extracted from it (cannabinoids) have gained widespread popularity. Moreover, sex differences have been proposed in epilepsy syndromes and in cannabinoid action. In the hippocampus, cannabinoids interact with the CB1R receptor whose membrane levels are regulated by β-Arrestin2, a protein that promotes its endocytosis and causes its downregulation. In this article, we evaluate the modulatory role of WIN 55,212-2 (WIN), a synthetic exogenous cannabinoid on behavioral convulsions and on the levels of CB1R and β-Arrestin2 in female and male adolescent rats after a single injection of the proconvulsant pentylenetetrazol (PTZ). As epilepsies can have a considerable impact on synaptic proteins that regulate neuronal toxicity, plasticity, and cognition, we also measured the levels of key proteins markers of excitatory synapses, in order to examine whether exogenous cannabinoids may prevent such pathologic changes after acute seizures. We found that the exogenous administration of WIN prevented convulsions of medium severity in females and males and increased the levels of phosphorylated CaMKII in the hippocampus. Furthermore, we observed a higher degree of colocalization between CB1R and β-Arrestin2 in the granule cell layer.

## Introduction

Epilepsy is a brain disorder caused by excessive neuronal activity, that may involve both (i.e., generalized) or only one (i.e., focal) hemisphere. Focal epilepsies usually involve the uncontrolled hyperexcitability of the hippocampal formation, a vulnerable region prone to developing seizures (Tatum, 2012; Chatzikonstantinou, 2014). If antiepileptic drugs (AEDs) do not improve the condition of patients (~30% of cases), resective surgery is the only remaining option, though this alternative presents its own challenges. Not all patients are surgical candidates, ~1/3 of patients will not be seizure free after the procedure, and comorbidities, including memory decline, may arise (Wiebe and Jette, 2012). Patients with drug-resistant epilepsies may be therefore drawn to try alternative treatments, including the smoking or consumption of the plant Cannabis sativa (commonly known as marihuana) or compounds derived from it, sometimes based on information obtained from the press or the internet (Kerr et al., 2019). Most preclinical epilepsy research has been focused on the two main cannabinoids found in the plant, Δ^9^-tetrahydrocannabinol and cannabidiol (THC and CBD, respectively), and on synthetic agonists of cannabinoid receptors 1 and 2 (CB1R and CB2R, respectively). In the brain, exogenous and endogenous cannabinoids exert much of their action through activation of presynaptic CB1R, a seven transmembrane G-protein coupled receptor (GPCR) that is present in most cell types, resulting in decreased neurotransmitter release through a Gi/o signaling pathway (Benarroch, 2014). CB2R is found at low levels in the CNS, though recent reports show that it is inducible in some pathologies (including epilepsy; Ji et al., 2021) and may modulate plasticity (Chen et al., 2017; Kendall and Yudowski, 2017). In the hippocampus, the majority of CB1R expression occurs in gamma-aminobutyric acid (GABA)-releasing neurons, mostly on the axon terminals of cholecystokinin containing interneurons (Katona et al., 1999; Marsicano and Lutz, 1999; Nyíri et al., 2005; Földy et al., 2006). Despite lower expression levels of this receptor in glutamatergic cells of the hippocampus (Katona et al., 2006; Kawamura et al., 2006), its activation results in a higher G protein coupled signaling efficiency (Steindel et al., 2013; Busquets-Garcia et al., 2018) compared to that of interneurons. CB1R also activates β-arrestin 1 (βarr1) and 2 (βarr2) after ligand-induced phosphorylation of the receptor by GPCR kinases, a process that is independent of G-protein signaling. βarr2 initiates the endocytosis of CB1R by binding to clathrin and causing an overall desensitization of the CB1R-mediated response, thus decreasing the response to endo- or exo cannabinoids (Nogueras-Ortiz and Yudowski, 2016).

Most studies and anecdotical reports have concluded that plant-derived or synthetic exogenous cannabinoids are generally anti-convulsive (Devinsky et al., 2014; Rosenberg et al., 2015, 2017; Kerr et al., 2019), though pro-convulsive effects have also been observed for CB1R agonists, such as THC (Malyshevskaya et al., 2017), WIN (Perescis et al., 2020), or arachidonyl-2’-chloroethylamide, ACEA (Vilela et al., 2013; for recent reviews see Smolyakova et al., 2020; Kaczor et al., 2022). Little is known about whether sex differences may exist in the therapeutic action of exogenous cannabinoids in the context of epilepsy, though behavioral changes (e.g., appetite, pain) due to cannabinoid actions do show sex differences in humans and animals (Cooper and Craft, 2018). This may be an important area of research, as sex differences have been reported as well on epilepsy types, their underlying mechanisms, and associated comorbidities (Christian et al., 2020). In this study, we hypothesized that activation of CB1Rs by exogenous cannabinoids leads to a reduction in the intensity of acutely-induced seizures that is more pronounced in females compared to males, due to lower βArr2 expression levels.

Furthermore, it is not known whether exogenous cannabinoids can mitigate the impact that chronic or acute seizures have on the levels of synaptic proteins that are important mediators of excitation, cognition, and/or neurotoxicity (e.g., postsynaptic density protein 95, PSD-95; GluA2 subunit of α-amino-3-hydroxy-5-methyl-4-isoxazolepropionic acid receptors, AMPAR; Ca^2+^/calmodulin-dependent protein kinase II, CaMKII) (Bronstein et al., 1990; Dong and Rosenberg, 2004; Ying et al., 2004; Needs et al., 2019; Lee and Kim, 2020). We therefore investigated whether similar changes could manifest early on, i.e., after an acute dose of PTZ, considered a “gold standard” in the evaluation of AEDs (Bialer and White, 2010), and whether underlying sex differences may be present.

Last, as WIN 55,212-2 (WIN from now on) is a CB1R and CB2R agonist (Felder et al., 1995), we used (±)-WIN 55,212, a racemic mixture consisting of WIN combined with the neutral CB2 antagonist, (−)-WIN 55,212-3, as a first approximation to evaluate the impact that activating CB1R while antagonizing CB2R could have in our acute model of PTZ-induced seizures.

## Materials and Methods

**Animals.** Adolescent female and male Sprague–Dawley rats (45–55 days old) were used, as this is one of the periods where the prevalence of epilepsy and years of life lost peaks (Beghi et al., 2019). All procedures involving animals were in accordance with the Bioethics Committee of the Universidad de los Andes (Las Condes, Chile).

**Drugs.** Pentylenetetrazol, PTZ (#P6500, Sigma-Aldrich, USA), 1-(2,4-dichlorophenyl)-5-(4-iodophenyl)-4-methyl-N-1-piperidinyl-1H-pyrazole-3-carboxamide (AM251, a CB1R antagonist), [(3R)-2,3-dihydro-5-methyl-3-(4-morpholinylmethyl) pyrrolo[1,2,3-de]-1,4-benzoxazin-6-yl]-1-naphthalenyl-methanone, monomethanesulfonate ((+)-WIN 55,212-2 (mesylate), WIN in the present article), [2,3-dihydro-5-methyl-3-(4-morpholinylmethyl)pyrrolo [1,2,3-de]-1,4-benzoxazin-6-yl]-1-naphthalenyl-methanone, methanesulfonate(±)-WIN 55,212 (mesylate; WINr in the present article), and N-(Piperidin-1-yl)-5-(4-iodophenyl)-1-(2,4-dichlorophenyl)-4-methyl-1H-pyrazole-3-carboxamide (AM251 in the present article), were obtained from Cayman chemical, USA (#71670, #10009023, and #10736, respectively).

**Injection Protocol.** The experimental design is shown in Supplementary Figure 1A. Drugs (or vehicles, i.e., DMSO or 0.9% NaCl) were injected i.p. WIN dose used was 1 mg/kg, a dose previously shown to be neuroprotective and anticonvulsive in juvenile rats (Rudenko et al., 2012) while PTZ was 50 mg/kg. One hour after the last injection animals were either euthanized for Western blot analysis or perfused for immunohistochemistry.

**Euthanasia.** Rats were euthanized by rapid decapitation (Holson, 1992; van Rijn et al., 2011).

**Seizure Activity.** The four groups of animals were: C, WIN, PTZ, and WIN+PTZ, either male (*n* = 4, 4, 7, 4, respectively); or female (*n* = 9, 10, 17, 12) were video recorded simultaneously for 1 h after a single PTZ (or corresponding vehicle) injection. Offline behavioral analysis by a blind observer was done for the initial 25 min, as this is when maximal effects were observed. The time of onset, duration, and severity of seizures were described according to a modified Racine scale for rats (Lüttjohann et al., 2009). This scale evaluates intensity stages, going from 1 (lowest severity, e.g., motionless stare) to 6 (highest severity, e.g., strong convulsions).

**Cellular Fractioning.** The separation of proteins from crude membrane extracts and cytosol was accomplished as previously detailed (Carlin et al., 1980; Wyneken et al., 2001). Briefly, brains were quickly removed after decapitation, the hippocampi extracted, flash-frozen in liquid nitrogen, and stored at −80°C until the day of homogenization. The latter was performed in icecold homogenization buffer; 0.32 M sucrose, 5 mM Tris-HCl, 0.5 mM EGTA pH 7.4, along with protease (cOmplete^TM^ Protease Inhibitor Cocktail #11697498001, Sigma-Aldrich, USA) and phosphatase inhibitors (Phosphatase Inhibitor Cocktail B1500, Bimake, USA), using a Teflon homogenizer. A P1 pellet was obtained after an initial 10 min centrifugation at 1,000 *g*. The resultant supernatant (supernatant A) was kept in ice while the pellet was resuspended in homogenization buffer and centrifuged at 700 *g* for 10 min. The resultant pellet (cellular debris) was discarded, and the supernatant added to supernatant A, and this mix was centrifuged at 12,000 *g* for 20 min. This supernatant was stored as the “cytoplasm” fraction and the pellet was resuspended in radioimmunoprecipitation assay (RIPA) buffer with protease inhibitors (the “membrane” fraction). Protein concentration was determined by the bicinchoninic acid (BCA) method of protein quantification using bovine serum albumin (BSA) as the standard. Sample absorbance was read in a Tecan Infinite M1000 PRO microplate reader (Tecan, Switzerland).

Western blots were performed using standard methodology as previously described (Lafourcade et al., 2020). Primary antibodies used were anti CB1, 1:1,000 (Ab23703, Abcam, UK), anti βArr2, 1:500 (Ab31294, Abcam), anti phospho T286phosphorylated calcium/calmodulin-dependent protein kinase II (p-CaMKII), 1:2,000 (Ab32678, Abcam), anti postsynaptic density protein 95 (PSD95) 1:1,000 (#610496, BD Biosciences, USA), anti α-amino-3-hydroxy-5-methyl-4-isoxazolepropionic acid, AMPA, subunit GluA2 (GluA2), 1:1,000 (Sc-517265, Santa Cruz Biotechnology, USA), anti calcium/calmodulin-dependent protein kinase II (total CaMKII) (Sc-5306, Santa Cruz Biotechnology), antiGolgi matrix protein 130 (GM130), 1:1,000 (#610823, BD Biosciences, USA), anti βIII-Tubulin, 1:2,500 (G7121, Promega, USA), anti N-cadherin, 1:1,000 (#33-3900, Invitrogen). Secondary antibodies were obtained from Li-cor, USA (#926-80010, #926-80011) and Invitrogen, USA (#81-1620).

**Perfusion.** Animals were anesthetized by an i.p. injection of ketamine (100 mg/kg) and xylazine (30 mg/kg). A standard protocol of intracardial perfusion was performed (Gage et al., 2012), first with ~200 ml of phosphate-buffered saline (PBS, pH = 7.4), followed by ~400 ml of paraformaldehyde 4% (PFA) to fix brain tissue. Perfusion was done at a steady rate of ~20 ml/minute using a Masterflex Pump, model 7518 (Masterflex^®^ Bioprocessin, USA).

**Immunofluorescence.** After perfusion animals were decapitated, brains removed, and post-fixed in PFA overnight (ON) followed by sucrose 30% in PBS until decantation. Brains were embedded in optimal cutting temperature compound (OCT) and sectioned (30 μm coronal sections) using a cryostat (Microm HM 525, Thermo Fisher Scientific). Sections were washed in PBS and permeabilized with Triton-X 0.2%: PBS for 30 min. Tissue was then incubated in a blocking solution containing 5% BSA and 5% horse serum in PBS for 45 min. Primary antibodies used were GluA2, 1:800 (sc-517265, Santa Cruz Biotechnology), CB1R, 1:900 (ab23703, Abcam), βArr2, 1:400 (ab31294, Abcam), and Cleaved-Cas3 (CAS3), 1:600 (9661, Cell Signaling); diluted in antibody solution (1% BSA and 1% horse serum in PBS), and incubated overnight at 4°C with agitation. Brain sections were then washed with Tween 0.2% in PBS three times (20 min each time), followed by incubation witha secondary antibody for 90 min. After three washes with PBS, sections were mounted using mounting media with DAPI and analyzed by confocal microscopy (Leica Sp8). For each condition/treatment, a group of at least five different sections were examined in the hippocampal region at Bregma -3.6 to -4.5 mm (Kruger et al., 1995; Cambridge University Press). Images were edited by Adobe Photoshop (CC 2014, California, USA) to normalize intra and inter sample background across all different channels. The average fluorescence intensities of four randomly placed sample areas (2,500 μm^2^) in the granule cell layer or the hilus was used to calculate nominal (e.g., CB1R+) or colocalization (e.g., CB1R + βArr2) values. The area of reactivity of each antibody were normalized to the size of the sampled area.

**Statistics.**

Two-tailed *t*-tests and ANOVA and outlier analysis tests were conducted in GraphPad Prism version 6, GraphPad Software, San Diego, California USA. MANOVA tests were performed in R Core Team (2020). R: A language and environment for statistical computing. R Foundation for Statistical Computing, Vienna, Austria.

## Results

**Figure 1:**
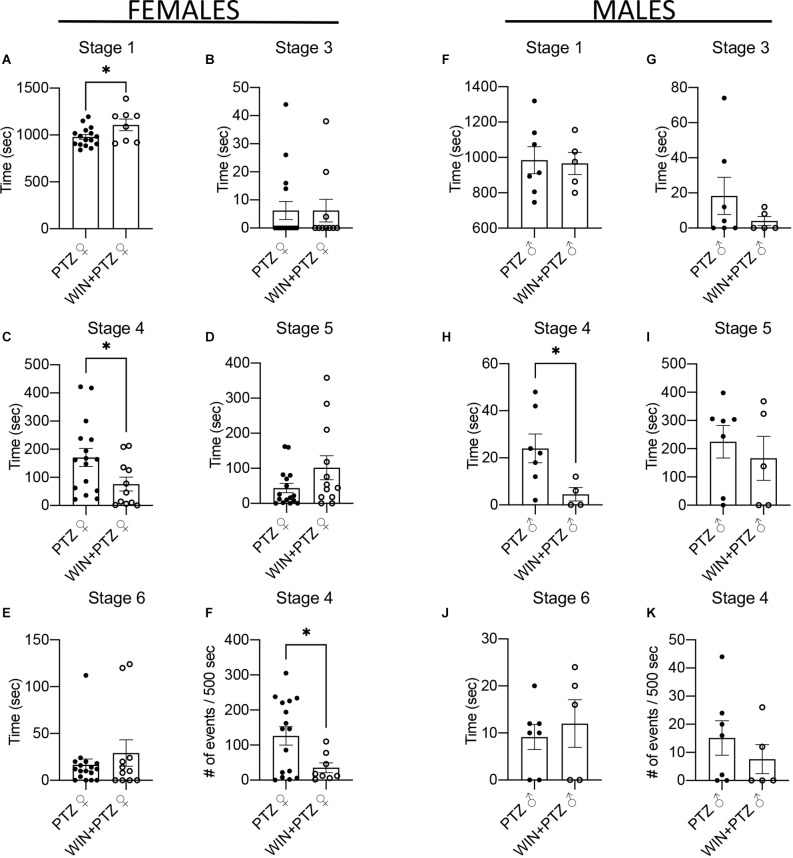
Effect of WIN on PTZ-induced convulsions. **(A–E)** Seizure duration in females (PTZ: *n* = 16 for stages 1, 3, 4, 5; and *n* = 17 for stage 6. WIN + PTZ: *n* = 8, 10, 11, 12, 11 for stages 1, 3, 4, 5, 6, respectively). **(F)** Number of times that female rats reach stage 4 in the first 500 s (PTZ *n* = 9, WIN + PTZ, *n* = 8). **(F–J)** Seizure duration in males (PTZ: *n* = 7 for all stages. WIN + PTZ: *n* = 5 for stages 1, 3, 5, 6; *n* = 4 for stage 4). **(K)** Number of times that male rats reach stage 4 in the first 500 s (PTZ *n* = 7, WIN + PTZ, *n* = 5). ^*^p < 0.05, *t*-test.

First, we evaluated the effect of a single injection of WIN 55,212-2 (WIN from now on) on grooming and locomotion. Female and male rats injected with WIN showed no differences in the time spent grooming (Supplementary Figures 1B,C), ambulating (Supplementary Figures 1D,E), or motionless (Supplementary Figures 1F,G; Supplementary Table 1). To examine the effect of exogenous cannabinoids on acute convulsions we first injected female or male rats with either a vehicle (i.e., DMSO) or with WIN, and an hour later with either a vehicle (i.e., NaCl 0.9%) or Pentylenetetrazole (PTZ; Supplementary Figure 1A). We then compared the time of convulsions using a modified Racine scale at each stage of severity (Lüttjohann et al., 2009; Figures 1A–J; Supplementary Table 1). Animals not injected with PTZ (control group, C, and WIN group) were recorded but not included in the analysis, as they did not display convulsions. We found a significant increase in the duration females spent at stage 1 (e.g., behavioral arrest) in animals injected with WIN + PTZ (Figure 1A), and a significant decrease in the time females and males spent in stage 4 (clonic seizures, sitting, Figures 1C,H, respectively). A MANOVA test that included males and females confirmed a significant effect for treatment and sex at stage 4 (Supplementary Table 1). The time spent at stage 2 (e.g., facial jerking) is not shown as few animals showed this behavior. No significant differences were found for stages 3 (e.g., neck jerks, Figure 1B, females and Figure 1G, males), 5 (convulsions while lying down, Figure 1D, females and Figure 1I, males) or 6 (e.g severe convulsions, including jumping, Figure 1E, females and Figure 1J, males). The WIN-mediated significant reduction of time spent on stage 4 convulsions (Figure 1C, females and Figure 1H, males) was abolished in females (Supplementary Figure 1H) and males (Supplementary Figure 1I; Supplementary Table 1) when the CB1R antagonist AM251 was injected 30 min before WIN, indicating that the anticonvulsive effect of the latter was mediated by CB1R. In our hands hormonal changes did not have a strong effect on acute PTZ-induced convulsions in the presence or absence of WIN, as we did not find significant differences between the time spent at higher stages of seizure severity (i.e., stages 4, 5, and 6) when considering the estrous cycle period in females (Supplementary Figure 1J, Supplementary Table 1).

**Figure 2:**
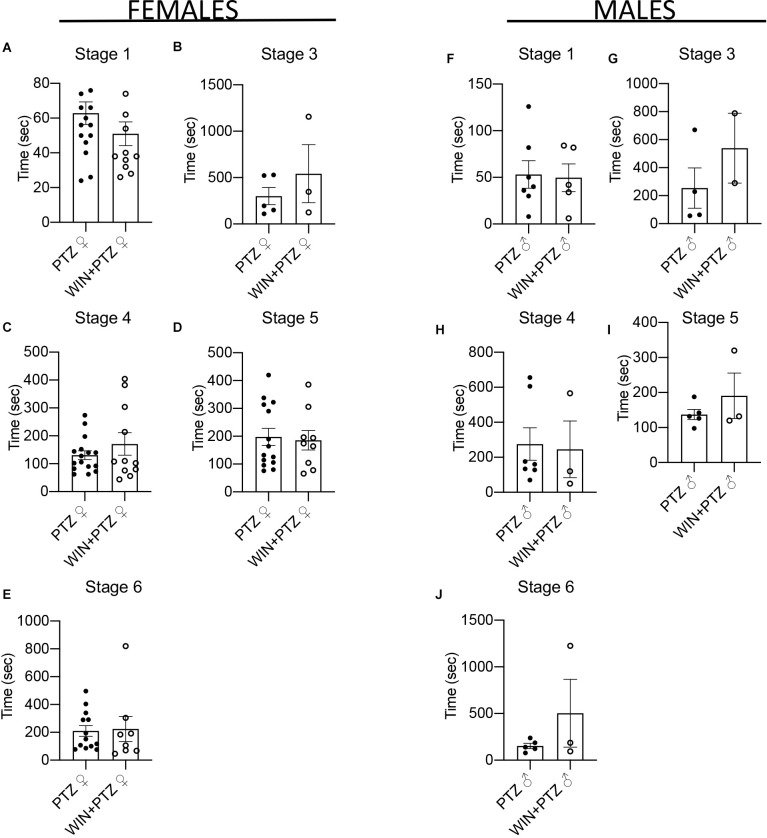
Latency to epileptic crisis at stages of increasing severity. **(A–E)** Latency in females (PTZ: *n* = 16 for stages 1 and 4, *n* = 5 for stage 3, *n* = 14 for stage 5, *n* = 13 for stage 6. WIN + PTZ: *n* = 12, 11, 9, and 8 for stages 1, 4, 5, 6, respectively, *n* = 3 for stage 3). **(F–J)** Latency to epileptic crisis at stages of increasing severity in males (PTZ: *n* = 7 stages 1 and 4; *n* = 4 for stage 3, *n* = 5 for stages 5 and 6. WIN + PTZ: *n* = 5 for stage 1, *n* = 2 for stage 3, *n* = 3 for stages 4, 5, and 6).

We calculated the number of times animals reached stage 4 in the first 500 s of recording, as this was the time when generally most crises were recorded (Supplementary Figures 2A–F). Females injected with PTZ showed a significant increase in the number of times they reached stage 4 compared to those injected with WIN + PTZ (Figure 1F). No differences between these groups were observed in males (Figure 1J). No differences were observed when considering other time periods (i.e., from 500 to 1,000 s, or from 1,000 to 1,500 s (Supplementary Table 1). No significant differences were observed in the latency to reach each intensity stage (Figures 2A–E, females and Figures 2F–J, males; Supplementary Table 2).

Since sex differences have been observed in the binding levels of βArr2 to the corticotropin-releasing factor (CRF) receptor during the stress response (Bangasser et al., 2010), we wondered if similar mechanisms could be found in our model. To answer this question, we performed Western blots from whole hippocampal tissue. To distinguish between membrane-bound (e.g., CB1R) or cytoplasmic (e.g., βArr2) proteins (Srivastava et al., 2015), we performed a differential centrifugation protocol after the homogenization of the samples (see “Materials and Methods” Section). After establishing that our centrifugation method yielded samples enriched in membrane-bound proteins (see Supplementary Methods, Supplementary Figures 3A–C), we compared the levels of CB1R between males and females in the membrane fraction. We did not obtain significant differences in hippocampal levels of CB1R in males compared to females (Supplementary Figures 3D,E; Supplementary Table 3), though a tendency toward higher levels of CB1R in males can be observed, which is consistent with previous results (Ferraro et al., 2020). No significant differences were found in the cytoplasmic levels of βArr2 between males and females either (Supplementary Figures 3F,G; Supplementary Table 3).

**Figure 3:**
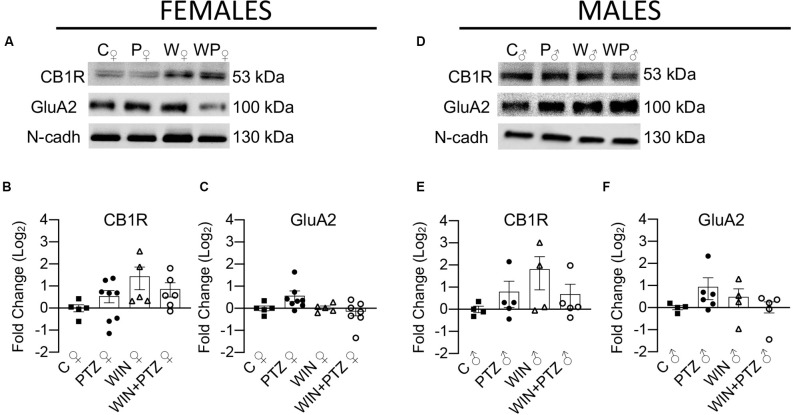
Expression levels of membrane-bound CB1R and AMPAR subunit GluA2 in females and males. **(A)** Representative Western blots of membrane-bound CB1R and GluA2, in females and **(D)** males. **(B)** Quantification of membrane-bound CB1R levels in females (C, *n* = 5; PTZ, *n* = 8; WIN, *n* = 5; WIN + PTZ, *n* = 6). **(C)** Quantification of membrane-bound GluA2 levels in females (C, *n* = 5; PTZ, *n* = 8; WIN, *n* = 5; WIN + PTZ, *n* = 7).** (E)** Quantification of membrane-bound CB1R levels in males (C, *n* = 4; PTZ, *n* = 5; WIN, *n* = 4; WIN + PTZ, *n* = 5). **(F)** Quantification of membrane-bound GluA2 levels in males (C, *n* = 4; PTZ, *n* = 6; WIN, *n* = 4; WIN + PTZ, *n* = 5). N-cadherin was used as protein loading control for all gels.

Next, we examined the impact of PTZ injection in the presence or absence of WIN on hippocampal levels of membrane-bound CB1R and GluA2, an AMPAR subunit that is important for neuronal synaptic plasticity and memory processes (Hara et al., 2012). We did not observe significant changes in the levels of these two proteins, either in samples obtained from females (Figures 3A–C) or males (Figures 3D–F; Supplementary Table 3).

**Figure 4:**
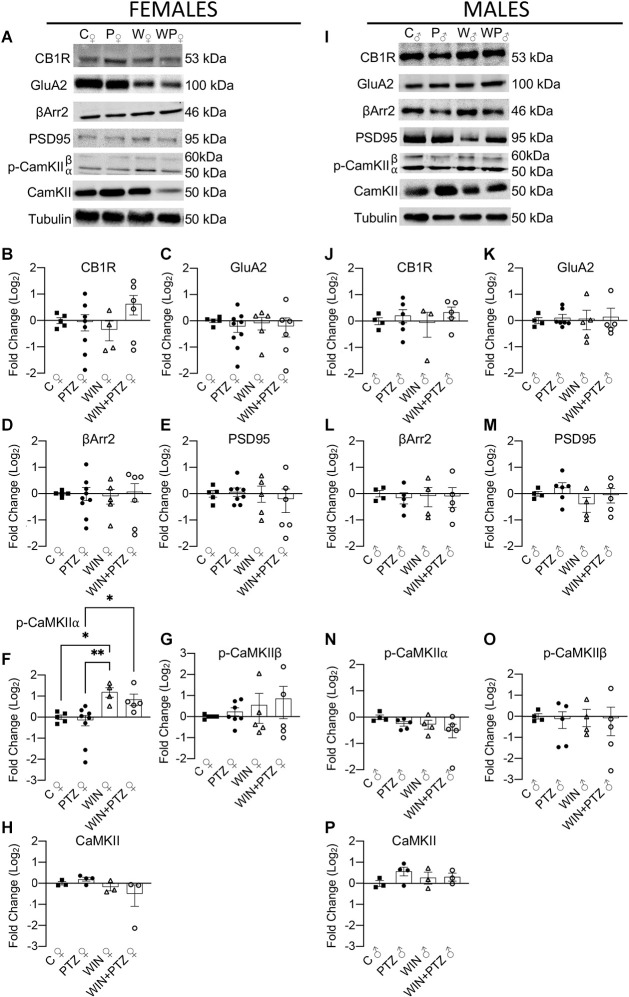
Expression levels of proteins obtained from a fraction enriched in cytoplasmic proteins/proteins bound to small organelles. **(A)** Representative Western blots of CB1R, GluA2R, βArr2, PSD95, p-CaMKII and total CaMKII in females and **(I)** males. **(B–H)** Quantification of **(A)** (Females. CB1R: C, *n* = 5; PTZ, *n* = 8; WIN, *n* = 4; WIN + PTZ, *n* = 6; GluA2: C, *n* = 5; PTZ, *n* = 9; WIN, *n* = 5; WIN + PTZ, *n* = 6; βArr2: C, *n* = 5; PTZ, *n* = 9; WIN, *n* = 5; WIN + PTZ, *n* = 6; PSD95: C, *n* = 5; PTZ, *n* = 8; WIN, *n* = 5; WIN + PTZ, *n* = 6, *p*-CaMKIIα: C, *n* = 5; PTZ, *n* = 8; WIN, *n* = 4; WIN + PTZ, *n* = 5; *p*-CaMKIIβ: C, *n* = 5; PTZ, *n* = 7; WIN, *n* = 5; WIN + PTZ, *n* = 5; total CaMKII: C, *n* = 3; PTZ, *n* = 4; WIN, *n* = 3; WIN + PTZ, *n* = 3). **(J–P)** Quantification of **(D)** (Males. CB1R: C, *n* = 4; PTZ, *n* = 6; WIN, *n* = 3; WIN + PTZ, *n* = 5; GluA2: C, *n* = 4; PTZ, *n* = 7; WIN, *n* = 5; WIN + PTZ, *n* = 5; βArr2: C, *n* = 4; PTZ, *n* = 5; WIN, *n* = 4; WIN + PTZ, *n* = 5; PSD95: C, *n* = 4; PTZ, *n* = 6; WIN, *n* = 4; WIN + PTZ, *n* = 5, *p*-CaMKIIα: C, *n* = 4; PTZ, *n* = 5; WIN, *n* = 4; WIN + PTZ, *n* = 5; *p*-CaMKIIβ: C, *n* = 4; PTZ, *n* = 5; WIN, *n* = 4; WIN + PTZ, *n* = 5; total CaMKII: C, *n* = 3; PTZ, *n* = 4; WIN, *n* = 3; WIN + PTZ, *n* = 3). Tubulin was used as protein loading control for all gels. ^*^p < 0.05 and ^**^p < 0.01, one-way ANOVA with Tukey’s multiple comparisons *post hoc*.

Similarly, we evaluated whether the levels of cytoplasmic proteins relevant for CB1R action (e.g., βArr2) and synaptic efficiency (i.e., GluA2, PSD95, and CaMKII) were altered after an acute exposure to PTZ, previously injected with WIN or vehicle (Figure 4). No differences were found in the levels of CB1R or GluA2 in our “cytoplasmic” fraction between groups, either in females (Figures 4A–C; Supplementary Table 4) or males (Figures 4I–K; Supplementary Table 4). We also measured the expression levels of βArr2 and PSD95, and did not observe differences in their expression levels, either in females (Figures 4A,D,E) or males (Figures 4I,L,M; Supplementary Table 4). We did observe a significant increase in the levels of phosphorylated CaMKIIα in the WIN group compared to the control and PTZ groups, and also between the WIN + PTZ group and the PTZ group in females (Figures 4A,F), but not in males (Figures 4I,N; Supplementary Table 4). No significant differences were obtained between groups in females or males when comparing the levels of *p*-CaMKIIβ (Figures 4A,G,I,O, respectively) or total CaMKII (Figures 4A,H,I,P, respectively; Supplementary Table 4).

**Figure 5:**
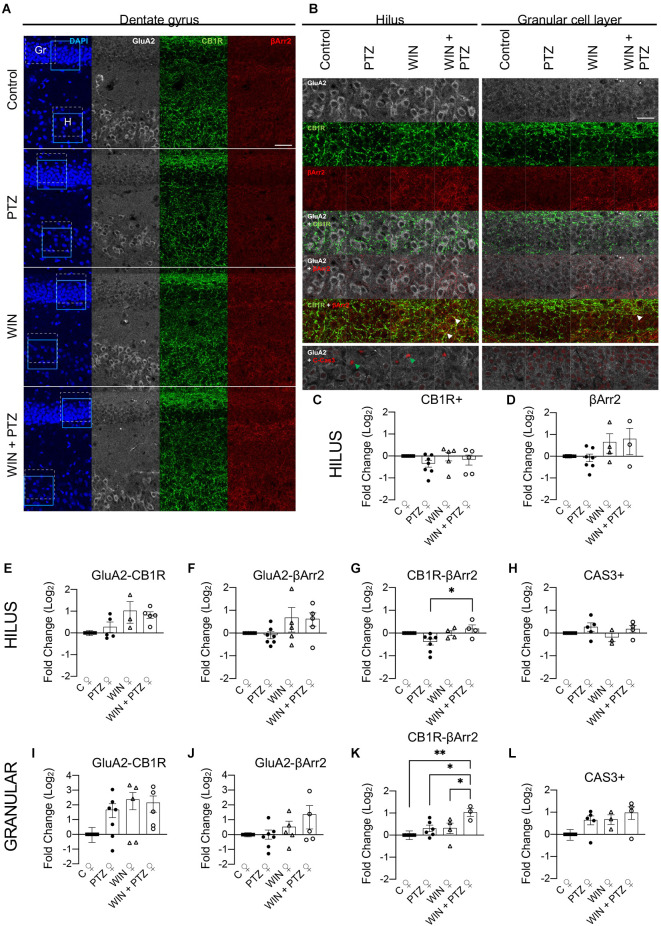
Hilar and granule celllayer immunohistochemistry in female rats. **(A)**Representative immunofluorescent images of the dentate gyrus showingthe granule cell layer (Gr) and the hilus (H) for each experimental group. Dashed square: areas used for colocalization analysis. Calibration bar: 40 μm. **(B)** Representative images used for analysis [amplified view of dashed square region from **(A)**]. Calibration bar: 25 μm. **(C–L)** quantification of **(B)**. **(C)** CB1R + (C, *n* = 6; PTZ, *n* = 7; WIN, *n* = 5; WIN + PTZ, *n* = 5) and **(D)** βArr2+ (C, *n* = 5; PTZ, *n* = 7; WIN, *n* = 4; WIN + PTZ, *n* = 3) area in the hilus. **(E)** GluA2-CB1R (C, *n* = 5; PTZ, *n* = 5; WIN, *n* = 3; WIN + PTZ, *n* = 5), **(F)** GluA2-βArr2 (C, *n* = 5; PTZ, *n* = 7; WIN, *n* = 5; WIN + PTZ, *n* = 5) and **(G)** CB1R-βArr2 (C, *n* = 5; PTZ, *n* = 7; WIN, *n* = 4; WIN + PTZ, *n* = 4) colocalization in hilus. **(H)** CAS3+ neurons (C, *n* = 4; PTZ, *n* = 5; WIN, *n* = 3; WIN + PTZ, *n* = 4) in hilus. **(I)** GluA2-CB1R (C, *n* = 5; PTZ, *n* = 7; WIN, *n* = 5; WIN + PTZ, *n* = 5), **(J)** GluA2-βArr2 (C, *n* = 5; PTZ, *n* = 7; WIN, *n* = 5; WIN + PTZ, *n* = 5), and **(K)** CB1R-βArr2 (C, *n* = 5; PTZ, *n* = 6; WIN, *n* = 4; WIN + PTZ, *n* = 3) colocalization in the granular cell layer. **(L)** CAS3+ neurons (C, *n* = 4; PTZ, *n* = 5; WIN, *n* = 3; WIN + PTZ, *n* = 4) in granular cell layer. White arrows: examples of strong colocalization between CB1R and βArr2. Green arrows: example of CAS3+ cells. ^*^p < 0.05 and ^**^p < 0.01, one-way ANOVA with Tukey’s multiple comparisons *post hoc*.

As regional changes in βArr2 and CB1R could be missed when studying the whole hippocampus, we analyzed whether putative differences in the expression levels of βArr2 and CB1R that were not visible by Western blot could be revealed by local examination of the hippocampal dentate gyrus of female rats (representative figures, Figures 5A,B; Supplementary Table 5). This region was chosen as it is crucial in the control of excitability arising from other brain areas (e.g., entorhinal cortex), though seizures may be generated regardless of external inputs (Krook-Magnuson, 2017). In the hilus of the dentate gyrus excitatory mossy cells express CB1R (Krook-Magnuson et al., 2015; Sugaya et al., 2016; Jensen et al., 2021). Thus, in order to differentiate glutamatergic mossy cells from GABAergic interneurons in this region, we used GluA2 as a marker of mossy cells (Leranth et al., 1996). We did not find differences between our experimental groups in the expression levels of CB1R or bArr2 regardless of cell type (Figures 5C,D respectively), or when considering CB1R or bArr2 expression in hilar mossy cells (i.e., g, gluA2+ neurons; Figures 5E,F, respectively), a result that is consistent with our whole hippocampal Western blots (Figures 3, 4). However, CB1R and βArr2 colocalization in the hilus (in GluA2+ regions) increased in the WIN + PTZ group when compared to PTZ (Figure 5G). Since neuronal loss has been observed in hippocampal tissue obtained from mesial temporal lobe patients and from chronic PTZ kindling models of epilepsy (Yardimoglu et al., 2007; Kitaura et al., 2018), we measured the levels of cleaved caspase-3 (CAS3), a marker of cells that are dying (Crowley and Waterhouse, 2016), in hippocampal slices from our experimental groups. We did not observe significant differences in the levels of CAS3 for any of the groups studied (Figure 5H). In the granule cell layer, GluA2 is a marker of mature granule cells (Hagihara et al., 2011), and colocalization of GluA2 with CB1R or βArr2 did not show differences between groups (Figures 5I,J, respectively). However, colocalization of CB1R with βArr2 in GluA2+ cells was increased in the WIN + PTZ group compared to the other conditions (Figure 5K). No differences in cellular apoptosis were found between groups in the granule cell layer (Figure 5L).

Finally, we examined the putative anticonvulsant effect of WIN55212 (WIN_r_), a racemic mixture of WIN 55,212-2 (WIN, the (+) isomer) and WIN 55,212-3 (the (-) isomer; Supplementary Figure 4; Supplementary Table 6). WIN 55,212-3 is considered an inactive enantiomer of WIN 55,212-2 due to the differences in potency between these two drugs (Savinainen et al., 2005). As WIN 55,212-3 is also a neutral antagonist of CB2R (Savinainen et al., 2005), we intended to investigate whether antagonizing CB2R, a receptor that has been implicated in epilepsy (Ji et al., 2021), could influence the anticonvulsive effect of WIN. Using the same protocol as previously mentioned, our results show only slight differences between the outcome of both treatments. This time WIN_r_ had anticonvulsive properties only on females, in stage 1 (Supplementary Figure 4A) and stage 5 (Supplementary Figure 4D) of the modified Racine scale. We did not observe a significant difference in any other stage. We performed a similar calculation as with Figure 1 and counted the number of times animals reached stage 5 in the first 500 s of recording (Supplementary Figures 6A–F). Females injected with PTZ showed a significant increase in the number of times they reached stage 5 compared to those injected with WIN + PTZ (Supplementary Figure 4F). No differences were observed in males (Supplementary Figure 4L).

No differences were observed in females or males when considering other time periods (i.e., from 500 to 1,000 s, or from 1,000 to 1,500 s (Supplementary Table 6).

No significant differences were found in the latency to reach the first convulsion for any stage of severity, in either females or males (Supplementary Figures 5A–H; Supplementary Table 7).

## Discussion

Our results show that treatment with WIN is effective in reducing medium-stage convulsion times (i.e., stage 4) in females and males injected with WIN previous to PTZ, while higher stages (i.e., 5 and 6) were not affected. We also observed a slight but significant increase in the time spent on stage 1 (e.g., motionless stare) in females injected with WIN followed by PTZ, which may compensate for the reduction in stage 4 seizures.

### Possible Antiepileptic Action of CB1R Agonists

Overall, this could be suggestive of cannabinoid agonists being an appropriate anticonvulsive therapy for patients expressing specific subtypes of epilepsy (e.g., complex-partial or focal onset impaired awareness epilepsy is associated with lower stages of the Racine scale, while higher stages represent generalized seizures; Englot and Blumenfeld, 2009; Van Erum et al., 2019) or cannabinoids being effective only in convulsions of a certain intensity, perhaps bearing some resemblance to what has been described for AED (Manford, 2017). Further experiments are needed to examine whether the interaction of WIN with AEDs that may be effective in treating seizures of higher severity could result in better anticonvulsive action. Discrepancies in the literature regarding the anticonvulsive properties of CB1R agonists may be due to differences in the experimental designs used. Moreover, a better comprehension of underlying molecular mechanisms is also necessary to reconcile results that show WIN having a proconvulsive (Vilela et al., 2013) or mixed (Wendt et al., 2011) action in preclinical models. In this regard, exploring the putative direct or indirect interactions between exogenous or endogenous CB1R agonists and receptors from other systems (e.g., transient receptor potential vanilloid 1, serotonin receptors, etc) may help explain the reasons behind the aforementioned discrepancies (Manna and Umathe, 2012; Carletti et al., 2016; Colangeli et al., 2019). Similarly, it would be interesting to evaluate in our model the impact of other compounds that have been previously shown to be successful in clinical or/and preclinical studies, like CBD (alone or combined with WIN). Promising results have been obtained with CBD in preclinical and clinical trials to treat genetic-based epilepsies, like Lennox-Gastaut Syndrome (LGS) and Dravet Syndrome (DS). This has led the Food and Drug Administration (FDA) to approve the use of Epidiolex, a drug based on purified CBD, to treat these two types of epilepsy (Silvestro et al., 2019). Similarly, positive modulators of CB1R have shown antiseizure properties in models of absence seizures (Roebuck et al., 2021; McElroy et al., 2022). It remains to be tested whether these compounds may interact with WIN to improve its anticonvulsive properties.

A limitation of our model is that we are focused on acute seizures, which may be viewed as a first step in the evaluation of CB1R agonists as anticonvulsants in females. Future experiments need to address whether regular administration of a CB1R agonist may be of use in models of epileptogenesis. This would require careful analysis of appropriate dosages and types of CB1R agonists, as adolescents exposed to synthetic cannabinoids (e.g., Spice/K2) present a higher incidence of seizures compared to those that are only exposed to cannabis (Havenon et al., 2011; Anderson et al., 2019).

### WIN-Induced Changes in Signaling Systems and Synaptic Proteins

Taking into consideration the similarities in our behavioral data between males and females, we analyzed whether their molecular strategies (i.e., changes in the expression levels of CB1R and the associated protein βArr2) in response to an acute PTZ treatment in the presence or absence of WIN were similar as well. Our results show there were no differences between our experimental groups in either females or males on hippocampal levels of membrane-bound CB1R or cytoplasmic βArr2, though the lack of changes in synaptic protein levels in our model may be due to insufficient time for those modifications to be discernible. Interestingly, the selective increase in p-CaMKII (α subunit) levels in the WIN + PTZ group in females (and not in males) compared to the PTZ one, and in WIN injected animals compared to PTZ-injected animals and control animals suggests that WIN may be triggering a synaptic remodeling (Lemieux et al., 2012). This may include remodeling of the cannabinoid signaling system involved in the reduction of seizure severity. Considering that mice that lack the α subunit (i.e., null mutants) of this enzyme show increased susceptibility to epileptic seizures (Butler et al., 1995), the increase in p-CaMKIIα we observe could be interpreted as a mechanism to control hyperexcitability through induction of plastic changes in glutamatergic synapses that should be further explored (Robison, 2014). It needs to be established whether WIN-induced plasticity in the PTZ group reduces seizures after a second or third PTZ challenge.

Since changes in the levels of CB1R or βArr2 may not be widespread, but rather localized to certain hippocampal regions, we analyzed the levels of these proteins in the dentate gyrus by immunohistochemistry.

The increase in the colocalization levels of CB1R with βArr2 firstly confirms that the aforementioned synaptic remodeling is on course. Moreover, the increased colocalization in regions that are GluA2+ suggests that the activity of CB1R in mossy cells is reduced in the presence of WIN due to a higher interaction of CB1R with βArr2. This could result in mossy cells having higher levels of activity (e.g., due to reduced inhibition by cannabinoids), a result that may seem counterintuitive under the “irritable” mossy cell hypothesis (i.e., mossy cells exert a net excitatory effect over granule cells), but not under the “dormant” hypothesis (i.e., mossy cells inhibit granule cells through activation of interneurons; Jinde et al., 2013). In that line, a recent publication supports the idea that an increase in mossy cell activity in chronic epileptic mice reduces the possibility of electrographic seizures from becoming behavioral ones (Bui et al., 2018).

### Possible Contribution of CB2R

Last, and given the discrepancies observed when examining the anticonvulsive effects of CB2R agonists and antagonists, we wondered whether the racemic mixture of (+) WIN 55,212-2 and (-) WIN 55,212-3 (WINr), could regulate convulsions differently than (+) WIN 55,212-2 alone. WIN 55,212-3 is a CB2 neutral antagonist/inverse agonist (Govaerts et al., 2004; Savinainen et al., 2005) capable of inhibiting the action of potent and specific CB2R inverse agonists (albeit at higher concentrations; Savinainen et al., 2005; Saroz et al., 2019). Our results with WIN_r_ were only slightly dissimilar to those obtained with WIN, implying that CB2R modulation could be a factor to consider when developing anticonvulsive drugs based on CB1R agonism. More research would be needed to examine the level of CB2R antagonism in the brain by our injection of WINr, as this study is limited by the low number of publications that have used this compound previously. Although it is necessary to expand these results using other available CB2R antagonists, our findings are partially consistent with the beneficial effects obtained by inhibiting CB2R (with a specific antagonist) in male rats (Rizzo et al., 2014).

## Conclusion

Taken together, our results suggest that, under an acute PTZ-induced seizure model, the cannabinoid agonist WIN has a significant effect on medium-stage seizure severity. This is associated with a specific increase in the phosphorylation of CaMKIIα, a central enzyme involved in the control of synaptic plasticity and neuronal excitability, and with an increase in the colocalization of CB1R with βarr2 in the granule cell layer of the dentate gyrus, showing that WIN is triggering plastic changes in the hippocampal region that is essential in the control of seizure activity.

## Data Availability Statement

The raw data supporting the conclusions of this article will be made available by the authors, without undue reservation.

## Ethics Statement

The animal study was reviewed and approved by Comité Ético Científico Universidad de los Andes.

## Author Contributions

AZR, MM-R, MG, BA, JC, SK, and CL carried out the experiments. CL and UW conceived the experiments and wrote the manuscript. AZR, MM-R, CL, AIJ, and UW contributed to the interpretation of results. AZR, CL, and MM-R designed the figures. CL, AZR, MM-R, and AIJ, analyzed the data. LR, UW, and CL contributed to the implementation of the research. All authors gave critical feedback and LR, UW, and CL revised the final manuscript. All authors contributed to the article and approved the submitted version.

## Conflict of Interest

The authors declare that the research was conducted in the absence of any commercial or financial relationships that could be construed as a potential conflict of interest.

## Publisher’s Note

All claims expressed in this article are solely those of the authors and do not necessarily represent those of their affiliated organizations, or those of the publisher, the editors and the reviewers. Any product that may be evaluated in this article, or claim that may be made by its manufacturer, is not guaranteed or endorsed by the publisher.

## References

[B1] Anderson S. A. R. Oprescu A. M. Calello D. P. Monte A. Dayan P. S. Hurd Y. L.. (2019). Neuropsychiatric sequelae in adolescents with acute synthetic cannabinoid toxicity. Pediatrics 144:e20182690. 10.1542/peds.2018-2690 31285395 PMC6697124

[B2] Bangasser D. A. Curtis A. Reyes B. A. S. Bethea T. T. Parastatidis I. Ischiropoulos H.. (2010). Sex differences in corticotropin-releasing factor receptor signaling and trafficking: potential role in female vulnerability to stress-related psychopathology. Mol. Psychiatry 15, 896–904. 10.1038/mp.2010.66 20548297 PMC2935505

[B3] Beghi E. Giussani G. Abd-Allah F. Abdela J. Abdelalim A. Abraha H. N.. (2019). Global, regional and national burden of epilepsy, 1990–2016: a systematic analysis for the Global Burden of Disease Study 2016. Lancet Neurol. 18, 357–375. 10.1016/S1474-4422(18)30454-X 30773428 PMC6416168

[B4] Benarroch E. E. (2014). Synaptic effects of cannabinoids: complexity, behavioral effects and potential clinical implications. Neurology 83, 1958–1967. 10.1212/WNL.0000000000001013 25339212

[B5] Bialer M. White H. S. (2010). Key factors in the discovery and development of new antiepileptic drugs. Nat. Rev. Drug Discov. 9, 68–82. 10.1038/nrd2997 20043029

[B6] Bronstein J. M. Farber D. B. Micevych P. E. Lasher R. Wasterlain C. G. (1990). Kindling induced changes in calmodulin kinase II immunoreactivity. Brain Res. 524, 49–53. 10.1016/0006-8993(90)90490-3 2169328

[B7] Bui A. D. Nguyen T. M. Limouse C. Kim H. K. Szabo G. G. Felong S.. (2018). Dentate gyrus mossy cells control spontaneous convulsive seizures and spatial memory. Science 359, 787–790. 10.1126/science.aan4074 29449490 PMC6040648

[B8] Busquets-Garcia A. Bains J. Marsicano G. (2018). CB 1 receptor signaling in the brain: extracting specificity from ubiquity. Neuropsychopharmacology 43, 4–20. 10.1038/npp.2017.206 28862250 PMC5719111

[B9] Butler L. S. Silva A. J. Abeliovich A. Watanabe Y. Tonegawa S. McNamara J. O. (1995). Limbic epilepsy in transgenic mice carrying a Ca^2+^/calmodulin-dependent kinase II α-subunit mutation. Proc. Natl. Acad. Sci. U S A 92, 6852–6855. 10.1073/pnas.92.15.6852 7624331 PMC41427

[B10] Carletti F. Gambino G. Rizzo V. Ferraro G. Sardo P. (2016). Involvement of TRPV1 channels in the activity of the cannabinoid WIN 55,212-2 in an acute rat model of temporal lobe epilepsy. Epilepsy Res. 122, 56–65. 10.1016/j.eplepsyres.2016.02.005 26970948

[B11] Carlin R. K. Grab D. J. Cohen R. S. Siekevitz P. (1980). Isolation and characterization of postsynaptic densities from various brain regions: enrichment of different types of postsynaptic densities. J. Cell Biol. 86, 831–845. 10.1083/jcb.86.3.831 7410481 PMC2110694

[B12] Chatzikonstantinou A. (2014). Epilepsy and the hippocampus. Hippocampus Clin. Neurosci. 34, 121–142. 10.1159/000356435 24777136

[B13] Chen D. J. Gao M. Gao F. F. Su Q. X. Wu J. (2017). Brain cannabinoid receptor 2: expression, function and modulation. Acta Pharmacol. Sin. 38, 312–316. 10.1038/aps.2016.149 28065934 PMC5342669

[B14] Christian C. A. Reddy D. S. Maguire J. Forcelli P. A. (2020). Sex differences in the epilepsies and associated comorbidities: implications for use and development of pharmacotherapies. Pharmacol. Rev. 72, 767–800. 10.1124/pr.119.017392 32817274 PMC7495340

[B15] Colangeli R. Di Maio R. Pierucci M. Deidda G. Casarrubea M. Di Giovanni G. (2019). Synergistic action of CB 1 and 5-HT 2B receptors in preventing pilocarpine-induced status epilepticus in rats. Neurobiol. Dis. 125, 135–145. 10.1016/j.nbd.2019.01.026 30716469

[B16] Cooper Z. D. Craft R. M. (2018). Sex-dependent effects of cannabis and cannabinoids: a translational perspective. Neuropsychopharmacology 43, 34–51. 10.1038/npp.2017.140 28811670 PMC5719093

[B17] Crowley L. C. Waterhouse N. J. (2016). Detecting cleaved caspase-3 in apoptotic cells by flow cytometry. Cold Spring Harb. Protoc. 2016, 958–962. 10.1101/pdb.prot087312 27803251

[B18] Devinsky O. Cilio M. R. Cross H. Fernandez-Ruiz J. French J. Hill C.. (2014). Cannabidiol: pharmacology and potential therapeutic role in epilepsy and other neuropsychiatric disorders. Epilepsia 55, 791–802. 10.1111/epi.12631 24854329 PMC4707667

[B19] Dong Y. Rosenberg H. C. (2004). Brief seizure activity alters Ca^2+^/calmodulin dependent protein kinase II dephosphorylation and subcellular distribution in rat brain for several hours. Neurosci. Lett. 357, 95–98. 10.1016/j.neulet.2003.11.069 15036583

[B20] Englot D. J. Blumenfeld H. (2009). Consciousness and Epilepsy: Why Are Complex-Partial Seizures Complex? Amsterdam, Netherlands: Elsevier. 10.1016/S0079-6123(09)17711-7 PMC2901990 19818900

[B21] Felder C. C. Joyce K. E. Briley E. M. Mansouri J. Mackie K. Blond O.. (1995). Comparison of the pharmacology and signal transduction of the human cannabinoid CB1 and CB2 receptors. Mol. Pharmacol. 48, 443–450. 7565624

[B22] Ferraro A. Wig P. Boscarino J. Reich C. G. (2020). Sex differences in endocannabinoid modulation of rat CA1 dendritic neurotransmission. Neurobiol. Stress 13:100283. 10.1016/j.ynstr.2020.100283 33344734 PMC7739177

[B23] Földy C. Neu A. Jones M. V. Soltesz I. (2006). Presynaptic, activity-dependent modulation of cannabinoid type 1 receptor-mediated inhibition of GABA release. J. Neurosci. 26, 1465–1469. 10.1523/JNEUROSCI.4587-05.2006 16452670 PMC6675496

[B24] Gage G. J. Kipke D. R. Shain W. (2012). Whole animal perfusion fixation for rodents. J. Vis. Exp. e3564. 10.3791/3564 22871843 PMC3476408

[B25] Govaerts S. J. Hermans E. Lambert D. M. (2004). Comparison of cannabinoid ligands affinities and efficacies in murine tissues and in transfected cells expressing human recombinant cannabinoid receptors. Eur. J. Pharm. Sci. 23, 233–243. 10.1016/j.ejps.2004.07.013 15489124

[B26] Hagihara H. Ohira K. Toyama K. Miyakawa T. (2011). Expression of the AMPA receptor subunits GluR1 and GluR2 is associated with granule cell maturation in the dentate gyrus. Front. Neurosci. 5:100. 10.3389/fnins.2011.00100 21927594 PMC3168919

[B27] Hara Y. Punsoni M. Yuk F. Sehwan Park C. Janssen W. G. M. Rapp P. R.. (2012). Synaptic distributions of GluA2 and PKMζ in the monkey dentate gyrus and their relationships with aging and memory. J. Neurosci. 32, 7336–7344. 10.1523/JNEUROSCI.0605-12.2012 22623679 PMC3391702

[B28] Havenon A. d. Chin B. Thomas K. C. Afra P. (2011). The Secret “Spice”: an undetectable toxic cause of seizure. Neurohospitalist 1, 182–186. 10.1177/1941874411417977 23983854 PMC3726077

[B29] Holson R. R. (1992). Euthanasia by decapitation: evidence that this technique produces prompt, painless unconsciousness in laboratory rodents. Neurotoxicol. Teratol. 14, 253–257. 10.1016/0892-0362(92)90004-t 1522830

[B30] Jensen K. R. Berthoux C. Nasrallah K. Castillo P. E. (2021). Multiple cannabinoid signaling cascades powerfully suppress recurrent excitation in the hippocampus. Proc. Natl. Acad. Sci. U S A 118:e2017590118. 10.1073/pnas.2017590118 33468648 PMC7848601

[B31] Ji X. Zeng Y. Wu J. (2021). The CB2 receptor as a novel therapeutic target for epilepsy treatment. Int. J. Mol. Sci. 22:8961. 10.3390/ijms22168961 34445666 PMC8396521

[B32] Jinde S. Zsiros V. Nakazawa K. (2013). Hilar mossy cell circuitry controlling dentate granule cell excitability. Front. Neural Circuits 7:14. 10.3389/fncir.2013.00014 23407806 PMC3569840

[B33] Kaczor E. E. Greene K. Zacharia J. Tormoehlen L. Neavyn M. Carriero S. (2022). The potential proconvulsant effects of cannabis: a scoping review. J. Med. Toxicol. 10.1007/s13181-022-00886-3[Online ahead of print] 35352276 PMC9198115

[B34] Katona I. Sperlágh B. Sík A. Käfalvi A. Vizi E. S. Mackie K.. (1999). Presynaptically located CB1 cannabinoid receptors regulate GABA release from axon terminals of specific hippocampal interneurons. J. Neurosci. 19, 4544–4558. 10.1523/JNEUROSCI.19-11-04544.1999 10341254 PMC6782612

[B35] Katona I. Urbán G. M. Wallace M. Ledent C. Jung K. M. Piomelli D.. (2006). Molecular composition of the endocannabinoid system at glutamatergic synapses. J. Neurosci. 26, 5628–5637. 10.1523/JNEUROSCI.0309-06.2006 16723519 PMC1698282

[B36] Kawamura Y. Fukaya M. Maejima T. Yoshida T. Miura E. Watanabe M.. (2006). The CB1 cannabinoid receptor is the major cannabinoid receptor at excitatory presynaptic sites in the hippocampus and cerebellum. J. Neurosci. 26, 2991–3001. 10.1523/JNEUROSCI.4872-05.2006 16540577 PMC6673964

[B37] Kendall D. A. Yudowski G. A. (2017). Cannabinoid receptors in the central nervous system: their signaling and roles in disease. Front. Cell. Neurosci. 10:294. 10.3389/fncel.2016.00294 28101004 PMC5209363

[B38] Kerr A. Walston V. Wong V. S. S. Kellogg M. Ernst L. (2019). Marijuana use among patients with epilepsy at a tertiary care center. Epilepsy Behav. 97, 144–148. 10.1016/j.yebeh.2019.05.037 31252269 PMC7608609

[B39] Kitaura H. Shirozu H. Masuda H. Fukuda M. Fujii Y. Kakita A. (2018). Pathophysiological characteristics associated with epileptogenesis in human hippocampal sclerosis. EBioMedicine 29, 38–46. 10.1016/j.ebiom.2018.02.013 29478873 PMC5925580

[B40] Krook-Magnuson E. (2017). The gate and the source? The dentate gyrus takes central stage in temporal lobe epilepsy. Epilepsy Curr. 17, 48–49. 10.5698/1535-7511-17.1.48 28331472 PMC5340558

[B41] Krook-Magnuson E. Armstrong C. Bui A. Lew S. Oijala M. Soltesz I. (2015). *in vivo* evaluation of the dentate gate theory in epilepsy. J. Physiol. 593, 2379–2388. 10.1113/JP270056 25752305 PMC4457198

[B500] Kruger L. Saporta S. Swanson L. W.. (1995). Photographic Atlas of the Rat Brain: The Cell and Fiber Architecture Illustrated in Three Planes with Stereotaxic Coordinates. New York, NY: Cambridge University Press.

[B42] Lafourcade C. A. Fernández A. Ramírez J. P. Corvalán K. Carrasco M. Á. Iturriaga A.. (2020). A role for mir-26a in stress: a potential sEV biomarker and modulator of excitatory neurotransmission. Cells 9:1364. 10.3390/cells9061364 32492799 PMC7349773

[B43] Lee D. S. Kim J. E. (2020). PDI-mediated reduction of disulfide bond on PSD95 increases spontaneous seizure activity by regulating NR2A-PSD95 interaction in epileptic rats independent of S-nitrosylation. Int. J. Mol. Sci. 21:2094. 10.3390/ijms21062094 32197489 PMC7139850

[B44] Lemieux M. Labrecque S. Tardif C. Labrie-Dion É. LeBel É. De Koninck P. (2012). Translocation of CaMKII to dendritic microtubules supports the plasticity of local synapses. J. Cell Biol. 198, 1055–1073. 10.1083/jcb.201202058 22965911 PMC3444784

[B45] Leranth C. Szeidemann Z. Hsu M. Buzsáki G. (1996). AMPA receptors in the rat and primate hippocampus: a possible absence of GluR2/3 subunits in most interneurons. Neuroscience 70, 631–652. 10.1016/s0306-4522(96)83003-x 9045077

[B46] Lüttjohann A. Fabene P. F. van Luijtelaar G. (2009). A revised Racine’s scale for PTZ-induced seizures in rats. Physiol. Behav. 98, 579–586. 10.1016/j.physbeh.2009.09.005 19772866

[B47] Malyshevskaya O. Aritake K. Kaushik M. K. Uchiyama N. Cherasse Y. Kikura-Hanajiri R.. (2017). Natural (Δ9-THC) and synthetic (JWH-018) cannabinoids induce seizures by acting through the cannabinoid CB1 receptor. Sci. Rep. 7:10516. 10.1038/s41598-017-10447-2 28874764 PMC5585372

[B48] Manford M. (2017). Recent advances in epilepsy. J. Neurol. 264, 1811–1824. 10.1007/s00415-017-8394-2 28120042 PMC5533817

[B49] Manna S. S. S. Umathe S. N. (2012). Involvement of transient receptor potential vanilloid type 1 channels in the pro-convulsant effect of anandamide in pentylenetetrazole-induced seizures. Epilepsy Res. 100, 113–124. 10.1016/j.eplepsyres.2012.02.003 22386872

[B50] Marsicano G. Lutz B. (1999). Expression of the cannabinoid receptor CB1 in distinct neuronal subpopulations in the adult mouse forebrain. Eur. J. Neurosci. 11, 4213–4225. 10.1046/j.1460-9568.1999.00847.x 10594647

[B51] McElroy D. L. Roebuck A. J. Greba Q. Garai S. Brandt A. L. Yilmaz O.. (2022). The type 1 cannabinoid receptor positive allosteric modulators GAT591 and GAT593 reduce spike-and-wave discharges in Genetic Absence Epilepsy Rats from Strasbourg. IBRO Neurosci. Rep. 12, 121–130. 10.1016/j.ibneur.2022.01.006 35128516 PMC8804275

[B52] Needs H. I. Henley B. S. Cavallo D. Gurung S. Modebadze T. Woodhall G.. (2019). Changes in excitatory and inhibitory receptor expression and network activity during induction and establishment of epilepsy in the rat Reduced Intensity Status Epilepticus (RISE) model. Neuropharmacology 158:107728. 10.1016/j.neuropharm.2019.107728 31356824 PMC6892273

[B53] Nogueras-Ortiz C. Yudowski G. A. (2016). The multiple waves of cannabinoid 1 receptor signaling. Mol. Pharmacol. 90, 620–626. 10.1124/mol.116.104539 27338082 PMC11037448

[B54] Nyíri G. Cserép C. Szabadits E. Mackie K. Freund T. F. (2005). CB1 cannabinoid receptors are enriched in the perisynaptic annulus and on preterminal segments of hippocampal GABAergic axons. Neuroscience 136, 811–822. 10.1016/j.neuroscience.2005.01.026 16344153

[B55] Perescis M. F. J. Flipsen N. A. R. van Luijtelaar G. van Rijn C. M. (2020). Altered SWD stopping mechanism in WAG/Rij rats subchronically treated with the cannabinoid agonist R(+)WIN55,212-2. Epilepsy Behav. 102:106722. 10.1016/j.yebeh.2019.106722 31855784

[B56] Rizzo V. Carletti F. Gambino G. Schiera G. Cannizzaro C. Ferraro G.. (2014). Role of CB2 receptors and cGMP pathway on the cannabinoid-dependent antiepileptic effects in an *in vivo* model of partial epilepsy. Epilepsy Res. 108, 1711–1718. 10.1016/j.eplepsyres.2014.10.001 25458534

[B57] Robison A. J. (2014). Emerging role of CaMKII in neuropsychiatric disease. Trends Neurosci. 37, 653–662. 10.1016/j.tins.2014.07.001 25087161

[B58] Roebuck A. J. Greba Q. Smolyakova A. M. Alaverdashvili M. Marks W. N. Garai S.. (2021). Positive allosteric modulation of type 1 cannabinoid receptors reduces spike-and-wave discharges in Genetic Absence Epilepsy Rats from Strasbourg. Neuropharmacology 190:108553. 10.1016/j.neuropharm.2021.108553 33845076

[B59] Rosenberg E. C. Patra P. H. Whalley B. J. (2017). Therapeutic effects of cannabinoids in animal models of seizures, epilepsy, epileptogenesis and epilepsy-related neuroprotection. Epilepsy Behav. 70, 319–327. 10.1016/j.yebeh.2016.11.006 28190698 PMC5651410

[B60] Rosenberg E. C. Tsien R. W. Whalley B. J. Devinsky O. (2015). Cannabinoids and epilepsy. Neurotherapeutics 12, 747–768. 10.1007/s13311-015-0375-5 26282273 PMC4604191

[B61] Rudenko V. Rafiuddin A. Leheste J. R. Friedman L. K. (2012). Inverse relationship of cannabimimetic (R+)WIN 55,212 on behavior and seizure threshold during the juvenile period. Pharmacol. Biochem. Behav. 100, 474–484. 10.1016/j.pbb.2011.10.005 22019959

[B62] Saroz Y. Kho D. T. Glass M. Graham E. S. Grimsey N. L. (2019). Cannabinoid receptor 2 (CB2) signals via G-alpha-s and induces IL-6 and IL-10 cytokine secretion in human primary leukocytes. ACS Pharmacol. Transl. Sci. 2, 414–428. 10.1021/acsptsci.9b00049 32259074 PMC7088898

[B63] Savinainen J. R. Kokkola T. Salo O. M. H. Poso A. Järvinen T. Laitinen J. T. (2005). Identification of WIN55212-3 as a competitive neutral antagonist of the human cannabinoid CB 2 receptor. Br. J. Pharmacol. 145, 636–645. 10.1038/sj.bjp.0706230 15852035 PMC1576178

[B64] Silvestro S. Mammana S. Cavalli E. Bramanti P. Mazzon E. (2019). Use of cannabidiol in the treatment of epilepsy: efficacy and security in clinical trials. Molecules 24:1459. 10.3390/molecules24081459 31013866 PMC6514832

[B65] Smolyakova A. M. Zagzoog A. Brandt A. L. Black T. Mohamed K. Laprairie R. B. (2020). The endocannabinoid system and synthetic cannabinoids in preclinical models of seizure and epilepsy. J. Clin. Neurophysiol. 37, 15–27. 10.1097/WNP.0000000000000633 31895186

[B66] Srivastava A. Gupta B. Gupta C. Shukla A. K. (2015). Emerging functional divergence of β-arrestin isoforms in GPCR function. Trends Endocrinol. Metab. 26, 628–642. 10.1016/j.tem.2015.09.001 26471844

[B67] Steindel F. Lerner R. Häring M. Ruehle S. Marsicano G. Lutz B.. (2013). Neuron-type specific cannabinoid-mediated G protein signalling in mouse hippocampus. J. Neurochem. 124, 795–807. 10.1111/jnc.12137 23289830

[B68] Sugaya Y. Yamazaki M. Uchigashima M. Kobayashi K. Watanabe M. Sakimura K.. (2016). Crucial roles of the endocannabinoid 2-arachidonoylglycerol in the suppression of epileptic seizures. Cell Rep. 16, 1405–1415. 10.1016/j.celrep.2016.06.083 27452464

[B69] Tatum W. O. (2012). Mesial temporal lobe epilepsy. J. Clin. Neurophysiol. 29, 356–365. 10.1097/WNP.0b013e31826b3ab7 23027091

[B70] Van Erum J. Van Dam D. De Deyn P. P. (2019). PTZ-induced seizures in mice require a revised Racine scale. Epilepsy Behav. 95, 51–55. 10.1016/j.yebeh.2019.02.029 31026782

[B71] van Rijn C. M. Krijnen H. Menting-Hermeling S. Coenen A. M. L. (2011). Decapitation in rats: latency to unconsciousness and the “wave of death”. PLoS One 6:e16514. 10.1371/journal.pone.0016514 21304584 PMC3029360

[B72] Vilela L. R. Medeiros D. C. Rezende G. H. S. de Oliveira A. C. P. Moraes M. F. D. Moreira F. A. (2013). Effects of cannabinoids and endocannabinoid hydrolysis inhibition on pentylenetetrazole-induced seizure and electroencephalographic activity in rats. Epilepsy Res. 104, 195–202. 10.1016/j.eplepsyres.2012.11.006 23352737

[B73] Wendt H. Soerensen J. Wotjak C. T. Potschka H. (2011). Targeting the endocannabinoid system in the amygdala kindling model of temporal lobe epilepsy in mice. Epilepsia 52, e62–e65. 10.1111/j.1528-1167.2011.03079.x 21627644

[B74] Wiebe S. Jette N. (2012). Pharmacoresistance and the role of surgery in difficult to treat epilepsy. Nat. Rev. Neurol. 8, 669–677. 10.1038/nrneurol.2012.181 22964510

[B75] Wyneken U. Smalla K. H. Marengo J. J. Soto D. De la Cerda A. Tischmeyer W.. (2001). Kainate-induced seizures alter protein composition and N-methyl-D-aspartate receptor function of rat forebrain postsynaptic densities. Neuroscience 102, 65–74. 10.1016/s0306-4522(00)00469-3 11226670

[B76] Yardimoglu M. Ilbay G. Kokturk S. Onar F. D. Sahin D. Alkan F.. (2007). Light and electron microscopic examinations in the hippocampus of the rat brain following PTZ-induced epileptic seizures. J. Appl. Biol. Sci. 1, 97–106. Available online at: https://www.jabsonline.org/index.php/jabs/article/view/38.

[B77] Ying Z. Bingaman W. Najm I. M. (2004). Increased numbers of coassembled PSD-95 to NMDA-receptor subunits NR2B and NR1 in human epileptic cortical dysplasia. Epilepsia 45, 314–321. 10.1111/j.0013-9580.2004.37703.x 15030493

