# Supplementary material for: Behavioral and Molecular Responses to Exogenous Cannabinoids During Pentylenetetrazol-Induced Convulsions in Male and Female Rats
Source: Front Mol Neurosci. 2022 Aug 9;15:868583. doi: 10.3389/fnmol.2022.868583 (PMC9488559; doi:10.3389/fnmol.2022.868583)
Supplement: Supplementary file 1 [file Table_1.docx]

Supplementary Tables

| TABLE 1 |  |  |  |  |  |  |  |  |
| --- | --- | --- | --- | --- | --- | --- | --- | --- |
| **Time (sec) spent Grooming** | | | | |  |  |  |  |
|  | **Male ♂** | | **Female ♀** | |  |  |  |  |
|  |  |  |  |  |  |  |  |  |
|  | **PTZ** | **WIN+PTZ** | **PTZ** | **WIN+PTZ** |  |  |  |  |
| n | 4 | 4 | 10 | 10 |  |  |  |  |
| mean | 264.5 | 127 | 312.2 | 156 |  |  |  |  |
| SE | 76.28 | 38.51 | 87.48 | 31.25 |  |  |  |  |
| unpaired t-test |  | 0.1587 |  | 0.1099 |  |  |  |  |
|  |  |  |  |  |  |  |  |  |
| **Distance (m) travelled in 15 mins** | | | | |  |  |  |  |
|  | **Male ♂** | | **Female ♀** | |  |  |  |  |
|  |  |  | **PTZ** | **WIN+PTZ** |  |  |  |  |
| n | 4 | 4 | 7 | 7 |  |  |  |  |
| mean | 16 | 14 | 25.14 | 16.29 |  |  |  |  |
| SE | 7.314 | 4.848 | 4.945 | 3.617 |  |  |  |  |
| unpaired t-test |  | 0.8273 |  | 0.1738 |  |  |  |  |
|  |  |  |  |  |  |  |  |  |
| **Time (sec) spent motionless in 15 mins** | | | | |  |  |  |  |
|  | **Male ♂** | | **Female ♀** | |  |  |  |  |
|  |  |  | **PTZ** | **WIN+PTZ** |  |  |  |  |
| n | 4 | 4 | 6 | 6 |  |  |  |  |
| mean | 411.9 | 458.5 | 243.3 | 519.5 |  |  |  |  |
| SE | 127.4 | 67.59 | 102 | 88.7 |  |  |  |  |
| unpaired t-test |  | 0.758 |  | 0.0683 |  |  |  |  |
|  |  |  |  |  |  |  |  |  |
| **Time (sec) spent in each stage of epiletic crisis (increasing severity)** | | | | | |  |  |  |
| **Male ♂** | | | **Female ♀** | | |  |  |  |
| **Grade 1** | | | **Grade 1** | | |  |  |  |
|  | **PTZ** | **WIN+PTZ** |  | **PTZ** | **WIN+PTZ** |  |  |  |
| n | 7 | 5 |  | 16 | 8 |  |  |  |
| mean | 984.9 | 966 |  | 979.4 | 1108 |  |  |  |
| SE | 76.63 | 62.76 |  | 25.57 | 61.25 |  |  |  |
| unpaired t-test | | 0.8619 |  |  | 0.0316 |  |  |  |
|  |  |  |  |  | * |  |  |  |
| **Grade 3** | | | **Grade 3** | | |  |  |  |
|  | **PTZ** | **WIN+PTZ** |  | **PTZ** | **WIN+PTZ** |  |  |  |
| n | 7 | 5 |  | 16 | 10 |  |  |  |
| mean | 18.29 | 4 |  | 6.25 | 6.2 |  |  |  |
| SE | 10.62 | 2.53 |  | 3.188 | 4.049 |  |  |  |
| unpaired t-test | | 0.2947 |  |  | 0.9923 |  |  |  |
|  |  |  |  |  |  |  |  |  |
| **Grade 4** | | | **Grade 4** | | |  |  |  |
|  | **PTZ** | **WIN+PTZ** |  | **PTZ** | **WIN+PTZ** |  |  |  |
| n | 7 | 4 |  | 16 | 11 |  |  |  |
| mean | 24 | 4.5 |  | 169.5 | 76.18 |  |  |  |
| SE | 6.11 | 2.872 |  | 32.35 | 24.73 |  |  |  |
| unpaired t-test | | 0.0481 |  |  | 0.0449 |  |  |  |
|  |  | * |  |  | * |  |  |  |
| **Grade 5** | | | **Grade 5** | | |  |  |  |
|  | **PTZ** | **WIN+PTZ** |  | **PTZ** | **WIN+PTZ** |  |  |  |
| n | 7 | 5 |  | 16 | 12 |  |  |  |
| mean | 224.9 | 166 |  | 43.75 | 101.7 |  |  |  |
| SE | 57.62 | 77.99 |  | 13.46 | 34.34 |  |  |  |
| unpaired t-test | | 0.5478 |  |  | 0.095 |  |  |  |
|  |  |  |  |  |  |  |  |  |
| **Grade 6** | | | **Grade 6** | | |  |  |  |
|  | **PTZ** | **WIN+PTZ** |  | **PTZ** | **WIN+PTZ** |  |  |  |
| n | 7 | 5 |  | 17 | 11 |  |  |  |
| mean | 9.143 | 12 |  | 16.47 | 29.09 |  |  |  |
| SE | 2.685 | 5.06 |  | 6.258 | 14.08 |  |  |  |
| unpaired t-test | | 0.6007 |  |  | 0.3644 |  |  |  |
|  |  |  |  |  |  |  |  |  |
| **MANOVA** | | | | | |  |  |  |
|  |  |  |  |  |  |  |  |  |
| **Grade 1** | | | | | |  |  |  |
|  |  |  |  |  |  |  |  |  |
|  | Df | Sum Sq | Mean Sq | F value | Pr(>F) |  |  |  |
| Treatment | 1 | 16183 | 16183 | 0.7912 | 0.3813 |  |  |  |
| Sex | 1 | 19587 | 19587 | 0.9576 | 0.3362 |  |  |  |
| Residuals | 28 | 572706 | 20454 |  |  |  |  |  |
|  |  |  |  |  |  |  |  |  |
| **Grade 1** | | | | | |  |  |  |
|  |  | Sum Sq | Mean Sq | F value | e Pr(>F) |  |  |  |
| Treatment | 1 | 65287 | 65287 | 1.815 | 0.18924 |  |  |  |
| Sex | 1 | 118616 | 118616 | 3.2895 | 0.08046 |  |  |  |
| Residuals | 28 | 1009663 | 36059 |  |  |  |  |  |
|  |  |  |  |  |  |  |  |  |
| **Grade 4** | | | | | |  |  |  |
|  |  | Sum Sq | Mean Sq | F value | Pr(>F) |  |  |  |
| Treatment | 1 | 49759 | 49759 | 5.1073 | 0.03179 * |  |  |  |
| Sex | 1 | 122856 | 122856 | 12.6103 | 0.00138 ** |  |  |  |
| Residuals | 28 | 272791 | 9743 |  |  |  |  |  |
|  |  |  |  |  |  |  |  |  |
|  |  |  |  |  |  |  |  |  |
| **Grade 5** | | | | | |  |  |  |
|  |  | Sum Sq | Mean Sq | F value | Pr(>F) |  |  |  |
| Treatment | 1 | 10927 | 10927 | 0.7558 | 0.3920309 |  |  |  |
| Sex | 1 | 279907 | 279907 | 19.3619 | 0.0001425 *** |  |  |  |
| Residuals | 28 | 404785 | 14457 |  |  |  |  |  |
|  |  |  |  |  |  |  |  |  |
|  |  |  |  |  |  |  |  |  |
| **Grade 6** | | | | | |  |  |  |
|  |  | Sum Sq | Mean Sq | F value | e Pr(>F) |  |  |  |
| Treatment | 1 | 67.32 | 67.319 | 0.9477 | 0.3386 |  |  |  |
| Sex | 1 | 43.55 | 43.55 | 0.6131 | 0.4402 |  |  |  |
| Residuals | 28 | 1989 | 71.036 |  |  |  |  |  |
|  |  |  |  |  |  |  |  |  |
| **Grade 4 + AM251** | | | | | |  |  |  |
| **Male ♂** | | | | | |  |  |  |
|  |  | PTZ | WIN+PTZ | AM251+WIN+PTZ | |  |  |  |
| n |  | 3 | 3 | 2 |  |  |  |  |
| mean |  | 27.33 | 6 | 19 |  |  |  |  |
| SE |  | 7.424 | 3.464 | 13 |  |  |  |  |
| ANOVA |  |  |  | F (DFn, DFd) |  |  |  |  |
|  |  |  |  | F (2, 10) = 2.432 | |  |  |  |
|  |  |  |  | P=0.1378 |  |  |  |  |
|  |  |  |  |  |  |  |  |  |
| **Grade 4 + AM251s** | | | | | |  |  |  |
| **Female ♀** | | | | | |  |  |  |
|  |  | PTZ | WIN+PTZ | AM251+WIN+PTZ | |  |  |  |
| n |  | 5 | 3 | 3 |  |  |  |  |
| mean |  | 176.4 | 19.33 | 630 |  |  |  |  |
| SE |  | 33.95 | 13.53 | 409.1 |  |  |  |  |
| ANOVA |  |  |  | F (DFn, DFd) |  |  |  |  |
|  |  |  |  | F (2, 8) = 2.409 | |  |  |  |
|  |  |  |  | P=0.1517 |  |  |  |  |
|  |  |  |  |  |  |  |  |  |
| **Time on 4+ severity stage and EstrousCycle** | | | | | |  |  |  |
| **Female ♀** | | | | | |  |  |  |
|  | Estrus P | Estrus WP | Proestrus P | Proestrus WP |  |  |  |  |
| n | 3 | 10 | 3 | 3 |  |  |  |  |
| mean | 14 | 36.2 | 89.33 | 141.3 |  |  |  |  |
| SE | 4.163 | 15.42 | 74.6 | 78.32 |  |  |  |  |
|  | Metestrus P | Metestrus WP | Diestrus P | Diestrus WP |  |  |  |  |
| n | 12 | 3 | 21 | 12 |  |  |  |  |
| mean | 85.33 | 10.67 | 72.1 | 81 |  |  |  |  |
| SE | 34.92 | 5.457 | 24.32 | 31.14 |  |  |  |  |
| ANOVA |  |  |  | F (DFn, DFd) |  |  |  |  |
|  |  |  |  | F (7, 59) = 0.6979 | |  |  |  |
|  |  |  |  | P=0.6735 |  |  |  |  |
|  |  |  |  |  |  |  |  |  |
| **Number of times at stage 4 / 0-500 secs** | | | | | |  |  |  |
| **Male ♂** | | | | | |  |  |  |
|  | PTZ | WIN+PTZ |  |  |  |  |  |  |
| n | 6 | 5 |  |  |  |  |  |  |
| mean | 9 | 1.6 |  |  |  |  |  |  |
| SE | 3.821 | 1.166 |  |  |  |  |  |  |
| unpaired t-test |  | 0.1234 |  |  |  |  |  |  |
|  |  |  |  |  |  |  |  |  |
| **Number of times at stage 4 / 500-1000 secs** | | | | | |  |  |  |
| **Male ♂** | | | | | |  |  |  |
|  | PTZ | WIN+PTZ |  |  |  |  |  |  |
| n | 8 | 5 |  |  |  |  |  |  |
| mean | 7.5 | 1.6 |  |  |  |  |  |  |
| SE | 3.018 | 1.166 |  |  |  |  |  |  |
| unpaired t-test |  | 0.1667 |  |  |  |  |  |  |
|  |  |  |  |  |  |  |  |  |
| **Number of times at stage 4 / 1000-1500 secs** | | | | | |  |  |  |
| **Male ♂** | | | | | |  |  |  |
|  | PTZ | WIN+PTZ |  |  |  |  |  |  |
| n | 8 | 6 |  |  |  |  |  |  |
| mean | 0.25 | 0.3333 |  |  |  |  |  |  |
| SE | 0.25 | 0.3333 |  |  |  |  |  |  |
| unpaired t-test |  | 0.8414 |  |  |  |  |  |  |
|  |  |  |  |  |  |  |  |  |
| **Number of times at stage 4 / 0-500 secs** | | | | | |  |  |  |
| **Female ♂** | | | | | |  |  |  |
|  | PTZ | WIN+PTZ |  |  |  |  |  |  |
| n | 16 | 8 |  |  |  |  |  |  |
| mean | 126.4 | 35.5 |  |  |  |  |  |  |
| SE | 26.14 | 13.93 |  |  |  |  |  |  |
| unpaired t-test |  | 0.0279 |  |  |  |  |  |  |
|  |  | * |  |  |  |  |  |  |
| **Number of times at stage 4 / 500-1000 secs** | | | | | |  |  |  |
| **Female ♂** | | | | | |  |  |  |
|  | PTZ | WIN+PTZ |  |  |  |  |  |  |
| n | 16 | 9 |  |  |  |  |  |  |
| mean | 25.75 | 47.67 |  |  |  |  |  |  |
| SE | 13.99 | 34.67 |  |  |  |  |  |  |
| unpaired t-test |  | 0.4968 |  |  |  |  |  |  |
|  |  |  |  |  |  |  |  |  |
| **Number of times at stage 4 / 1000-1500 secs** | | | | | |  |  |  |
| **Female ♂** | | | | | |  |  |  |
|  | PTZ | WIN+PTZ |  |  |  |  |  |  |
| n | 16 | 9 |  |  |  |  |  |  |
| mean | 13 | 39.11 |  |  |  |  |  |  |
| SE | 12.48 | 25.4 |  |  |  |  |  |  |
| unpaired t-test |  | 0.31 |  |  |  |  |  |  |

| TABLE 2 |  |  |  |  |  |
| --- | --- | --- | --- | --- | --- |
| **Latency (sec) to reach each stage of epiletic crisis (increasing severity)** | | | | | |
| **Male ♂** | | | **Female ♀** | | |
| **Grade 1** | | | | | |
|  | **PTZ** | **WIN+PTZ** |  | **PTZ** | **WIN+PTZ** |
| n | 7 | 5 |  | 16 | 12 |
| mean | 53.14 | 49.6 |  | 62.88 | 51 |
| SE | 14.79 | 14.89 |  | 6.481 | 6.882 |
| unpaired t-test |  | 0.8731 |  |  | 0.2258 |
|  |  |  |  |  |  |
| **Grade 3** | | | | | |
|  | **PTZ** | **WIN+PTZ** |  | **PTZ** | **WIN+PTZ** |
| n | 4 | 2 |  | 5 | 3 |
| mean | 254.5 | 539 |  | 301.2 | 542.7 |
| SE | 144.1 | 249 |  | 92.38 | 313.4 |
| unpaired t-test |  | 0.3426 |  |  | 0.3887 |
|  |  |  |  |  |  |
| **Grade 4** | | | | | |
|  | **PTZ** | **WIN+PTZ** |  | **PTZ** | **WIN+PTZ** |
| n | 7 | 3 |  | 16 | 11 |
| mean | 275.7 | 245.3 |  | 130.5 | 170.9 |
| SE | 92.74 | 161.6 |  | 15.62 | 40.17 |
| unpaired t-test |  | 0.8669 |  |  | 0.2985 |
|  |  |  |  |  |  |
| **Grade 5** | | | | | |
|  | **PTZ** | **WIN+PTZ** |  | **PTZ** | **WIN+PTZ** |
| n | 5 | 3 |  | 14 | 9 |
| mean | 137.2 | 190.7 |  | 197.7 | 185.3 |
| SE | 14.65 | 64.76 |  | 30.71 | 34.96 |
| unpaired t-test |  | 0.3363 |  |  | 0.7969 |
|  |  |  |  |  |  |
| **Grade 6** | | | | | |
|  | **PTZ** | **WIN+PTZ** |  | **PTZ** | **WIN+PTZ** |
| n | 5 | 3 |  | 13 | 8 |
| mean | 153.2 | 503.3 |  | 210.3 | 223.8 |
| SE | 27.57 | 362.3 |  | 38.8 | 90.43 |
| unpaired t-test |  | 0.2379 |  |  | 0.8772 |
|  |  |  |  |  |  |
| **Time (sec) spent at stages 4 to 6 of seizure severity, according to estrous cycle** | | | | | |
| **Female ♀** | Estrus P | Estrus WP | Proestrus P | Proestrus WP | Metestrus P |
| n | 3 | 10 | 3 | 3 | 12 |
| mean | 14 | 36.2 | 89.33 | 141.3 | 85.33 |
| SE | 4.163 | 15.42 | 74.6 | 78.32 | 34.92 |
|  |  |  |  |  |  |
|  | Metestrus WP | Diestrus P | Diestrus WP |  |  |
| n | 3 | 21 | 12 |  |  |
| mean | 10.67 | 72.1 | 81 |  |  |
| SE | 5.457 | 24.32 | 31.14 |  |  |
| ANOVA |  |  |  | F (DFn, Fd) |  |
|  |  |  |  | F (7, 59) = 0.6979 | |
| P value |  |  |  | P=0.6735 |  |
|  |  |  |  |  |  |
|  |  |  |  |  |  |
|  |  |  |  |  |  |
|  |  |  |  |  |  |
|  |  |  |  |  |  |
|  |  |  |  |  |  |

| TABLE 3 |  |  |  |  |  |
| --- | --- | --- | --- | --- | --- |
| **N-cadherin** | | |  |  |  |
|  | Female ♀ | |  |  |  |
|  | Membrane | Cytoplasm |  |  |  |
| n | 4 | 4 |  |  |  |
| mean | 2.719 | 0.4485 |  |  |  |
| SE | 0.08665 | 0.1454 |  |  |  |
| unpaired t-test |  | <0.0001 |  |  |  |
|  |  | **** |  |  |  |
| **GM130** | | |  |  |  |
|  | Female ♀ | |  |  |  |
|  | Membrane | Cytoplasm |  |  |  |
| n | 4 | 4 |  |  |  |
| mean | 1.929 | 0.8829 |  |  |  |
| SE | 0.2736 | 0.1385 |  |  |  |
| unpaired t-test |  | 0.0143 |  |  |  |
|  |  | * |  |  |  |
| **Membrane** | | | | | |
| **CB1R** | | | | | |
|  | Female♀ | Male♂ |  |  |  |
|  | Membrane | Membrane |  |  |  |
| n | 5 | 4 |  |  |  |
| mean | 1 | 3.629 |  |  |  |
| SE | 0.3344 | 2.113 |  |  |  |
| unpaired t-test |  | 0.2077 |  |  |  |

| TABLE 3 (cont’d) | |  |  |  |  |  |  |  |  |  |  |  |  |  |  |  |  |  |  |  |
| --- | --- | --- | --- | --- | --- | --- | --- | --- | --- | --- | --- | --- | --- | --- | --- | --- | --- | --- | --- | --- |
| **CB1R/N-Cadherin** | | | | | |  |  |  |  |  |  |  |  |  |  |  |  |  |  |  |
|  |  | Female ♀ | |  |  |  |  |  |  |  |  |  |  |  |  |  |  |  |  |  |
|  | **C** | **PTZ** | **WIN** | **WIN+PTZ** |  |  |  |  |  |  |  |  |  |  |  |  |  |  |  |  |
| n | 5 | 8 | 5 | 6 |  |  |  |  |  |  |  |  |  |  |  |  |  |  |  |  |
| mean | 1.000 | 1.457 | 2.707 | 1.817 |  |  |  |  |  |  |  |  |  |  |  |  |  |  |  |  |
| SE | 0.1093 | 0.2788 | 0.9305 | 0.3980 |  |  |  |  |  |  |  |  |  |  |  |  |  |  |  |  |
| ANOVA |  |  |  |  | F (DFn, Fd) |  |  |  |  |  |  |  |  |  |  |  |  |  |  |  |
|  |  |  |  |  | F (3, 20) = 2.017 | |  |  |  |  |  |  |  |  |  |  |  |  |  |  |
| P value |  |  |  |  | P=0.1439 |  |  |  |  |  |  |  |  |  |  |  |  |  |  |  |
|  |  |  |  |  |  |  |  |  |  |  |  |  |  |  |  |  |  |  |  |  |
|  |  | Male ♂ | |  |  |  |  |  |  |  |  |  |  |  |  |  |  |  |  |  |
|  | **C** | **PTZ** | **WIN** | **WIN+PTZ** |  |  |  |  |  |  |  |  |  |  |  |  |  |  |  |  |
| n | 4 | 5 | 4 | 5 |  |  |  |  |  |  |  |  |  |  |  |  |  |  |  |  |
| mean | 1.000 | 1.734 | 3.513 | 1.605 |  |  |  |  |  |  |  |  |  |  |  |  |  |  |  |  |
| SE | 0.09984 | 0.6816 | 1.670 | 0.5868 |  |  |  |  |  |  |  |  |  |  |  |  |  |  |  |  |
| ANOVA |  |  |  |  | F (DFn, Fd) |  |  |  |  |  |  |  |  |  |  |  |  |  |  |  |
|  |  |  |  |  | F (3, 14) = 1.331 | |  |  |  |  |  |  |  |  |  |  |  |  |  |  |
| P value |  |  |  |  | P=0.3040 |  |  |  |  |  |  |  |  |  |  |  |  |  |  |  |
|  |  |  |  |  |  |  |  |  |  |  |  |  |  |  |  |  |  |  |  |  |
| **GluA2/ N-Cadherin** | | | | | |  |  |  |  |  |  |  |  |  |  |  |  |  |  |  |
|  |  | Female ♀ | |  |  |  |  |  |  |  |  |  |  |  |  |  |  |  |  |  |
|  | **C** | **PTZ** | **WIN** | **WIN+PTZ** |  |  |  |  |  |  |  |  |  |  |  |  |  |  |  |  |
| n | 5 | 8 | 5 | 7 |  |  |  |  |  |  |  |  |  |  |  |  |  |  |  |  |
| mean | 1.000 | 1.478 | 1.016 | 0.8997 |  |  |  |  |  |  |  |  |  |  |  |  |  |  |  |  |
| SE | 0.07828 | 0.2456 | 0.05907 | 0.1120 |  |  |  |  |  |  |  |  |  |  |  |  |  |  |  |  |
| ANOVA |  |  |  |  | F (DFn, Fd) |  |  |  |  |  |  |  |  |  |  |  |  |  |  |  |
|  |  |  |  |  | F (3, 21) = 2.547 | |  |  |  |  |  |  |  |  |  |  |  |  |  |  |
| P value |  |  |  |  | P=0.0834 |  |  |  |  |  |  |  |  |  |  |  |  |  |  |  |
|  |  |  |  |  |  |  |  |  |  |  |  |  |  |  |  |  |  |  |  |  |
|  |  | Male ♂ | |  |  |  |  |  |  |  |  |  |  |  |  |  |  |  |  |  |
|  | **C** | **PTZ** | **WIN** | **WIN+PTZ** |  |  |  |  |  |  |  |  |  |  |  |  |  |  |  |  |
| n | 4 | 6 | 4 | 5 |  |  |  |  |  |  |  |  |  |  |  |  |  |  |  |  |
| mean | 1.000 | 1.917 | 1.405 | 1.022 |  |  |  |  |  |  |  |  |  |  |  |  |  |  |  |  |
| SE | 0.06438 | 0.6306 | 0.4024 | 0.1759 |  |  |  |  |  |  |  |  |  |  |  |  |  |  |  |  |
| ANOVA |  |  |  |  | F (DFn, Fd) |  |  |  |  |  |  |  |  |  |  |  |  |  |  |  |
|  |  |  |  |  | F (3, 15) = 1.015 | |  |  |  |  |  |  |  |  |  |  |  |  |  |  |
| P value |  |  |  |  | P=0.4137 |  |  |  |  |  |  |  |  |  |  |  |  |  |  |  |

| TABLE 4 |  |  |  |  |  |  |  |  |  |  |  |  |  |  |  |  |  |  |  |  |
| --- | --- | --- | --- | --- | --- | --- | --- | --- | --- | --- | --- | --- | --- | --- | --- | --- | --- | --- | --- | --- |
| **CB1R/Tubulin** | | | | | |  |  |  |  |  |  |  |  |  |  |  |  |  |  |  |
|  |  | Female ♀ | |  |  |  |  |  |  |  |  |  |  |  |  |  |  |  |  |  |
|  | **C** | **PTZ** | **WIN** | **WIN+PTZ** |  |  |  |  |  |  |  |  |  |  |  |  |  |  |  |  |
| n | 5 | 8 | 4 | 6 |  |  |  |  |  |  |  |  |  |  |  |  |  |  |  |  |
| mean | 1 | 0.9632 | 0.7867 | 1.544 |  |  |  |  |  |  |  |  |  |  |  |  |  |  |  |  |
| SE | 0.07784 | 0.2045 | 0.2002 | 0.3893 |  |  |  |  |  |  |  |  |  |  |  |  |  |  |  |  |
| ANOVA |  |  |  |  | F (DFn, Fd) |  |  |  |  |  |  |  |  |  |  |  |  |  |  |  |
|  |  |  |  |  | F (3, 19) = 1,500 | |  |  |  |  |  |  |  |  |  |  |  |  |  |  |
| P value |  |  |  |  | P=0,2466 |  |  |  |  |  |  |  |  |  |  |  |  |  |  |  |
|  |  |  |  |  |  |  |  |  |  |  |  |  |  |  |  |  |  |  |  |  |
|  |  | Male ♂ | |  |  |  |  |  |  |  |  |  |  |  |  |  |  |  |  |  |
|  | **C** | **PTZ** | **WIN** | **WIN+PTZ** |  |  |  |  |  |  |  |  |  |  |  |  |  |  |  |  |
| n | 4 | 6 | 3 | 5 |  |  |  |  |  |  |  |  |  |  |  |  |  |  |  |  |
| mean | 1 | 1.154 | 0.9564 | 1.264 |  |  |  |  |  |  |  |  |  |  |  |  |  |  |  |  |
| SE | 0.09016 | 0.1957 | 0.303 | 0.1797 |  |  |  |  |  |  |  |  |  |  |  |  |  |  |  |  |
| ANOVA |  |  |  |  | F (DFn, Fd) |  |  |  |  |  |  |  |  |  |  |  |  |  |  |  |
|  |  |  |  |  | F (3, 14) = 0.4746 | |  |  |  |  |  |  |  |  |  |  |  |  |  |  |
| P value |  |  |  |  | P=0,7049 |  |  |  |  |  |  |  |  |  |  |  |  |  |  |  |
|  |  |  |  |  |  |  |  |  |  |  |  |  |  |  |  |  |  |  |  |  |
| **GluA2/tubulin** | | | | | |  |  |  |  |  |  |  |  |  |  |  |  |  |  |  |
|  |  | Female ♀ | |  |  |  |  |  |  |  |  |  |  |  |  |  |  |  |  |  |
|  | **C** | **PTZ** | **WIN** | **WIN+PTZ** |  |  |  |  |  |  |  |  |  |  |  |  |  |  |  |  |
| n | 5 | 9 | 5 | 6 |  |  |  |  |  |  |  |  |  |  |  |  |  |  |  |  |
| mean | 1 | 0.8611 | 0.9395 | 0.8661 |  |  |  |  |  |  |  |  |  |  |  |  |  |  |  |  |
| SE | 0.04399 | 0.1243 | 0.1548 | 0.2145 |  |  |  |  |  |  |  |  |  |  |  |  |  |  |  |  |
| ANOVA |  |  |  |  | F (DFn, Fd) |  |  |  |  |  |  |  |  |  |  |  |  |  |  |  |
|  |  |  |  |  | F (3, 21) = 0.1801 | |  |  |  |  |  |  |  |  |  |  |  |  |  |  |
| P value |  |  |  |  | P=0.9087 |  |  |  |  |  |  |  |  |  |  |  |  |  |  |  |
|  |  |  |  |  |  |  |  |  |  |  |  |  |  |  |  |  |  |  |  |  |
|  |  | Male ♂ | |  |  |  |  |  |  |  |  |  |  |  |  |  |  |  |  |  |
|  | **C** | **PTZ** | **WIN** | **WIN+PTZ** |  |  |  |  |  |  |  |  |  |  |  |  |  |  |  |  |
| n | 4 | 7 | 5 | 5 |  |  |  |  |  |  |  |  |  |  |  |  |  |  |  |  |
| mean | 1 | 1.077 | 1.049 | 1.106 |  |  |  |  |  |  |  |  |  |  |  |  |  |  |  |  |
| SE | 0.08006 | 0.09841 | 0.2623 | 0.2784 |  |  |  |  |  |  |  |  |  |  |  |  |  |  |  |  |
| ANOVA |  |  |  |  | F (DFn, Fd) |  |  |  |  |  |  |  |  |  |  |  |  |  |  |  |
|  |  |  |  |  | F (3, 17) = 0.04552 | |  |  |  |  |  |  |  |  |  |  |  |  |  |  |
| P value |  |  |  |  | P=0.9867 |  |  |  |  |  |  |  |  |  |  |  |  |  |  |  |
|  |  |  |  |  |  |  |  |  |  |  |  |  |  |  |  |  |  |  |  |  |
| **βArr2/tubulin** | | | | | |  |  |  |  |  |  |  |  |  |  |  |  |  |  |  |
|  |  | Female ♀ | |  |  |  |  |  |  |  |  |  |  |  |  |  |  |  |  |  |
|  | **C** | **PTZ** | **WIN** | **WIN+PTZ** |  |  |  |  |  |  |  |  |  |  |  |  |  |  |  |  |
| n | 5 | 9 | 5 | 6 |  |  |  |  |  |  |  |  |  |  |  |  |  |  |  |  |
| mean | 1 | 1.008 | 0.9372 | 1.054 |  |  |  |  |  |  |  |  |  |  |  |  |  |  |  |  |
| SE | 0.03216 | 0.1737 | 0.178 | 0.2482 |  |  |  |  |  |  |  |  |  |  |  |  |  |  |  |  |
| ANOVA |  |  |  |  | F (DFn, Fd) |  |  |  |  |  |  |  |  |  |  |  |  |  |  |  |
|  |  |  |  |  | F (3, 21) = 0.05608 | |  |  |  |  |  |  |  |  |  |  |  |  |  |  |
| P value |  |  |  |  | P=0.9821 |  |  |  |  |  |  |  |  |  |  |  |  |  |  |  |
|  |  |  |  |  |  |  |  |  |  |  |  |  |  |  |  |  |  |  |  |  |
|  |  | Male ♂ | |  |  |  |  |  |  |  |  |  |  |  |  |  |  |  |  |  |
|  | **C** | **PTZ** | **WIN** | **WIN+PTZ** |  |  |  |  |  |  |  |  |  |  |  |  |  |  |  |  |
| n | 4 | 5 | 4 | 5 |  |  |  |  |  |  |  |  |  |  |  |  |  |  |  |  |
| mean | 1 | 0.8925 | 0.94 | 0.9349 |  |  |  |  |  |  |  |  |  |  |  |  |  |  |  |  |
| SE | 0.08382 | 0.131 | 0.2328 | 0.2353 |  |  |  |  |  |  |  |  |  |  |  |  |  |  |  |  |
| ANOVA |  |  |  |  | F (DFn, Fd) |  |  |  |  |  |  |  |  |  |  |  |  |  |  |  |
|  |  |  |  |  | F (4, 15) = 0.05323 | |  |  |  |  |  |  |  |  |  |  |  |  |  |  |
| P value |  |  |  |  | P=0.9941 |  |  |  |  |  |  |  |  |  |  |  |  |  |  |  |
|  |  |  |  |  |  |  |  |  |  |  |  |  |  |  |  |  |  |  |  |  |
| **PSD95/tubulin** | | | | | |  |  |  |  |  |  |  |  |  |  |  |  |  |  |  |
|  |  | Female ♀ | |  |  |  |  |  |  |  |  |  |  |  |  |  |  |  |  |  |
|  | **C** | **PTZ** | **WIN** | **WIN+PTZ** |  |  |  |  |  |  |  |  |  |  |  |  |  |  |  |  |
| n | 5 | 8 | 5 | 6 |  |  |  |  |  |  |  |  |  |  |  |  |  |  |  |  |
| mean | 1 | 1.047 | 1.011 | 0.8694 |  |  |  |  |  |  |  |  |  |  |  |  |  |  |  |  |
| SE | 0.08758 | 0.1138 | 0.2114 | 0.262 |  |  |  |  |  |  |  |  |  |  |  |  |  |  |  |  |
| ANOVA |  |  |  |  | F (DFn, Fd) |  |  |  |  |  |  |  |  |  |  |  |  |  |  |  |
|  |  |  |  |  | F (3, 20) = 0.2018 | |  |  |  |  |  |  |  |  |  |  |  |  |  |  |
| P value |  |  |  |  | P=0.8939 |  |  |  |  |  |  |  |  |  |  |  |  |  |  |  |
|  |  |  |  |  |  |  |  |  |  |  |  |  |  |  |  |  |  |  |  |  |
|  |  | Male ♂ | |  |  |  |  |  |  |  |  |  |  |  |  |  |  |  |  |  |
|  | **C** | **PTZ** | **WIN** | **WIN+PTZ** |  |  |  |  |  |  |  |  |  |  |  |  |  |  |  |  |
| n | 4 | 6 | 4 | 5 |  |  |  |  |  |  |  |  |  |  |  |  |  |  |  |  |
| mean | 1 | 1.173 | 0.7604 | 0.9638 |  |  |  |  |  |  |  |  |  |  |  |  |  |  |  |  |
| SE | 0.06336 | 0.162 | 0.1469 | 0.1802 |  |  |  |  |  |  |  |  |  |  |  |  |  |  |  |  |
| ANOVA |  |  |  |  | F (DFn, Fd) |  |  |  |  |  |  |  |  |  |  |  |  |  |  |  |
|  |  |  |  |  | F (3, 15) = 1,192 | |  |  |  |  |  |  |  |  |  |  |  |  |  |  |
| P value |  |  |  |  | P=0.3462 |  |  |  |  |  |  |  |  |  |  |  |  |  |  |  |
|  |  |  |  |  |  |  |  |  |  |  |  |  |  |  |  |  |  |  |  |  |
| **p-CamKII alpha/tubulin** | | | | | |  |  |  |  |  |  |  |  |  |  |  |  |  |  |  |
|  |  | Female ♀ | |  |  |  |  |  |  |  |  |  |  |  |  |  |  |  |  |  |
|  | **C** | **PTZ** | **WIN** | **WIN+PTZ** |  |  |  |  |  |  |  |  |  |  |  |  |  |  |  |  |
| n | 5 | 8 | 4 | 5 |  |  |  |  |  |  |  |  |  |  |  |  |  |  |  |  |
| mean | 1 | 0.9036 | 2.283 | 1.799 |  |  |  |  |  |  |  |  |  |  |  |  |  |  |  |  |
| SE | 0.07521 | 0.1507 | 0.3578 | 0.3379 |  |  |  |  |  |  |  |  |  |  |  |  |  |  |  |  |
| ANOVA |  |  |  |  | F (DFn, Fd) |  |  |  |  |  |  |  |  |  |  |  |  |  |  |  |
|  |  |  |  |  | F (3, 18) = 7.695 | |  |  |  |  |  |  |  |  |  |  |  |  |  |  |
| P value |  |  |  |  | P=0.0016 |  |  |  |  |  |  |  |  |  |  |  |  |  |  |  |
|  |  | Post Hoc (Tukey) | |  |  |  |  |  |  |  |  |  |  |  |  |  |  |  |  |  |
|  |  | C vs. WIN | | * | 0.011 |  |  |  |  |  |  |  |  |  |  |  |  |  |  |  |
|  |  | PTZ vs. WIN+PTZ | | * | 0.0414 |  |  |  |  |  |  |  |  |  |  |  |  |  |  |  |
|  |  | PTZ vs. WIN | | ** | 0.0028 |  |  |  |  |  |  |  |  |  |  |  |  |  |  |  |
|  |  |  |  |  |  |  |  |  |  |  |  |  |  |  |  |  |  |  |  |  |
|  |  | Male ♂ | |  |  |  |  |  |  |  |  |  |  |  |  |  |  |  |  |  |
|  | **C** | **PTZ** | **WIN** | **WIN+PTZ** |  |  |  |  |  |  |  |  |  |  |  |  |  |  |  |  |
| n | 4 | 5 | 4 | 5 |  |  |  |  |  |  |  |  |  |  |  |  |  |  |  |  |
| mean | 1 | 0.8397 | 0.8245 | 0.7044 |  |  |  |  |  |  |  |  |  |  |  |  |  |  |  |  |
| SE | 0.05855 | 0.04976 | 0.09586 | 0.1237 |  |  |  |  |  |  |  |  |  |  |  |  |  |  |  |  |
| ANOVA |  |  |  |  | F (DFn, Fd) |  |  |  |  |  |  |  |  |  |  |  |  |  |  |  |
|  |  |  |  |  | F (3, 14) = 1,793 | |  |  |  |  |  |  |  |  |  |  |  |  |  |  |
| P value |  |  |  |  | P=0.1947 |  |  |  |  |  |  |  |  |  |  |  |  |  |  |  |
|  |  |  |  |  |  |  |  |  |  |  |  |  |  |  |  |  |  |  |  |  |
| **p-CamKII beta/tubulin** | | | | | |  |  |  |  |  |  |  |  |  |  |  |  |  |  |  |
|  |  | Female ♀ | |  |  |  |  |  |  |  |  |  |  |  |  |  |  |  |  |  |
|  |  |  |  |  |  |  |  |  |  |  |  |  |  |  |  |  |  |  |  |  |
|  | **C** | **PTZ** | **WIN** | **WIN+PTZ** |  |  |  |  |  |  |  |  |  |  |  |  |  |  |  |  |
| n | 5 | 7 | 5 | 5 |  |  |  |  |  |  |  |  |  |  |  |  |  |  |  |  |
| mean | 1 | 1.182 | 1.471 | 1.817 |  |  |  |  |  |  |  |  |  |  |  |  |  |  |  |  |
| SE | 0.02903 | 0.1595 | 0.673 | 0.8807 |  |  |  |  |  |  |  |  |  |  |  |  |  |  |  |  |
| ANOVA |  |  |  |  | F (DFn, Fd) |  |  |  |  |  |  |  |  |  |  |  |  |  |  |  |
|  |  |  |  |  | F (3, 18) = 0.4620 | |  |  |  |  |  |  |  |  |  |  |  |  |  |  |
| P value |  |  |  |  | P=0.7123 |  |  |  |  |  |  |  |  |  |  |  |  |  |  |  |
|  |  |  |  |  |  |  |  |  |  |  |  |  |  |  |  |  |  |  |  |  |
|  |  | Male ♂ | |  |  |  |  |  |  |  |  |  |  |  |  |  |  |  |  |  |
|  | **C** | **PTZ** | **WIN** | **WIN+PTZ** |  |  |  |  |  |  |  |  |  |  |  |  |  |  |  |  |
| n | 4 | 5 | 4 | 5 |  |  |  |  |  |  |  |  |  |  |  |  |  |  |  |  |
| mean | 1 | 0.9166 | 0.9831 | 0.9395 |  |  |  |  |  |  |  |  |  |  |  |  |  |  |  |  |
| SE | 0.08806 | 0.2461 | 0.2715 | 0.4118 |  |  |  |  |  |  |  |  |  |  |  |  |  |  |  |  |
| ANOVA |  |  |  |  | F (DFn, Fd) |  |  |  |  |  |  |  |  |  |  |  |  |  |  |  |
|  |  |  |  |  | F (3, 14) = 0.01655 | |  |  |  |  |  |  |  |  |  |  |  |  |  |  |
| P value |  |  |  |  | P=0.9970 |  |  |  |  |  |  |  |  |  |  |  |  |  |  |  |
|  |  |  |  |  |  |  |  |  |  |  |  |  |  |  |  |  |  |  |  |  |
| **total CamKII/tubulin** | | | | | |  |  |  |  |  |  |  |  |  |  |  |  |  |  |  |
|  |  | Female ♀ | |  |  |  |  |  |  |  |  |  |  |  |  |  |  |  |  |  |
|  | **C** | **PTZ** | **WIN** | **WIN+PTZ** |  |  |  |  |  |  |  |  |  |  |  |  |  |  |  |  |
| n | 3 | 4 | 3 | 3 |  |  |  |  |  |  |  |  |  |  |  |  |  |  |  |  |
| mean | 1 | 1.145 | 0.8858 | 0.708 |  |  |  |  |  |  |  |  |  |  |  |  |  |  |  |  |
| SE | 0.05579 | 0.07233 | 0.1074 | 0.2402 |  |  |  |  |  |  |  |  |  |  |  |  |  |  |  |  |
| ANOVA |  |  |  |  | F (DFn, Fd) |  |  |  |  |  |  |  |  |  |  |  |  |  |  |  |
|  |  |  |  |  | F (3, 9) = 2.103 | |  |  |  |  |  |  |  |  |  |  |  |  |  |  |
| P value |  |  |  |  | P=0.1701 |  |  |  |  |  |  |  |  |  |  |  |  |  |  |  |
|  |  |  |  |  |  |  |  |  |  |  |  |  |  |  |  |  |  |  |  |  |
|  |  | Male ♂ | |  |  |  |  |  |  |  |  |  |  |  |  |  |  |  |  |  |
|  | **C** | **PTZ** | **WIN** | **WIN+PTZ** |  |  |  |  |  |  |  |  |  |  |  |  |  |  |  |  |
| n | 3 | 4 | 3 | 3 |  |  |  |  |  |  |  |  |  |  |  |  |  |  |  |  |
| mean | 1 | 1.484 | 1.208 | 1.234 |  |  |  |  |  |  |  |  |  |  |  |  |  |  |  |  |
| SE | 0.09833 | 0.207 | 0.2341 | 0.1607 |  |  |  |  |  |  |  |  |  |  |  |  |  |  |  |  |
| ANOVA |  |  |  |  | F (DFn, Fd) |  |  |  |  |  |  |  |  |  |  |  |  |  |  |  |
|  |  |  |  |  | F (3, 9) = 1.174 | |  |  |  |  |  |  |  |  |  |  |  |  |  |  |
| P value |  |  |  |  | P=0.3727 |  |  |  |  |  |  |  |  |  |  |  |  |  |  |  |
|  |  |  |  |  |  |  |  |  |  |  |  |  |  |  |  |  |  |  |  |  |
| **total CamKII/tubulin** | | | | | |  |  |  |  |  |  |  |  |  |  |  |  |  |  |  |
|  |  | Male ♂ | |  |  |  |  |  |  |  |  |  |  |  |  |  |  |  |  |  |
|  | **C** | **PTZ** | **WIN** | **WIN+PTZ** |  |  |  |  |  |  |  |  |  |  |  |  |  |  |  |  |
| n | 3 | 4 | 3 | 3 |  |  |  |  |  |  |  |  |  |  |  |  |  |  |  |  |
| mean | 1 | 1.484 | 1.208 | 1.234 |  |  |  |  |  |  |  |  |  |  |  |  |  |  |  |  |
| SE | 0.09833 | 0.207 | 0.2341 | 0.1607 |  |  |  |  |  |  |  |  |  |  |  |  |  |  |  |  |
| ANOVA |  |  |  |  | F (DFn, Fd) |  |  |  |  |  |  |  |  |  |  |  |  |  |  |  |
|  |  |  |  |  | F (3, 9) = 1.174 | |  |  |  |  |  |  |  |  |  |  |  |  |  |  |
| P value |  |  |  |  | P=0.3727 |  |  |  |  |  |  |  |  |  |  |  |  |  |  |  |

| TABLE 5 |  |  |  |  |  |
| --- | --- | --- | --- | --- | --- |
| **CB1 hilus** | | | | | |
|  | **C** | **PTZ** | **WIN** | **WIN+PTZ** |  |
| n | 6 | 7 | 5 | 5 |  |
| mean | 1 | 0.7884 | 0.9895 | 0.8908 |  |
| SE | 0.03913 | 0.08485 | 0.1353 | 0.1399 |  |
| ANOVA |  |  |  | F (DFn, DFd) |  |
|  |  |  |  | F (3, 18) = 1,001 | |
| P value |  |  |  | P=0,4149 |  |
| **βarr hilus** | | | | | |
|  | **C** | **PTZ** | **WIN** | **WIN+PTZ** |  |
| n | 5 | 7 | 4 | 3 |  |
| mean | 1 | 0.9556 | 1.572 | 1.742 |  |
| SE | 0.08715 | 0.1152 | 0.4768 | 0.6919 |  |
| ANOVA |  |  |  | F (DFn, DFd) |  |
|  |  |  |  | F (3, 15) = 1,621 | |
| P value |  |  |  | P=0,2266 |  |
| **GluR2 CB1 hilus** | | | | | |
|  | **C** | **PTZ** | **WIN** | **WIN+PTZ** |  |
| n | 5 | 5 | 3 | 5 |  |
| mean | 1 | 1.215 | 2.037 | 1.78 |  |
| SE | 0.1207 | 0.2011 | 0.6751 | 0.1834 |  |
| ANOVA |  |  |  | F (DFn, DFd) |  |
|  |  |  |  | F (3, 14) = 2,967 | |
| P value |  |  |  | P=0,0682 |  |
| **CB1 βArr hilus** | | | | | |
|  | **C** | **PTZ** | **WIN** | **WIN+PTZ** |  |
| n | 5 | 7 | 5 | 5 |  |
| mean | 1 | 1.252 | 1.304 | 2.382 |  |
| SE | 0.1603 | 0.2095 | 0.4731 | 1.225 |  |
| ANOVA |  |  |  | F (DFn, DFd) |  |
|  |  |  |  | F (3, 18) = 0,9344 | |
| P value |  |  |  | P=0,4445 |  |
| **CAS3 hilus** | | | | | |
|  | **C** | **PTZ** | **WIN** | **WIN+PTZ** |  |
| n | 4 | 5 | 3 | 4 |  |
| mean | 1 | 1.209 | 0.8791 | 1.136 |  |
| SE | 0.08915 | 0.1578 | 0.1127 | 0.1254 |  |
| ANOVA |  |  |  | F (DFn, DFd) |  |
|  |  |  |  | F (3, 12) = 1,132 | |
| P value |  |  |  | P=0,3754 |  |
| **GluA2 hilus** | | | | | |
|  | **C** | **PTZ** | **WIN** | **WIN+PTZ** |  |
| n | 4 | 7 | 3 | 4 |  |
| mean | 1 | 1.232 | 1.472 | 1.267 |  |
| SE | 0.1018 | 0.1304 | 0.1621 | 0.1743 |  |
| ANOVA |  |  |  | F (DFn, DFd) |  |
|  |  |  |  | F (3, 14) = 1,350 | |
| P value |  |  |  | P=0,2985 |  |
| **GluA2 CB1 granulares** | | | | | |
|  | **C** | **PTZ** | **WIN** | **WIN+PTZ** |  |
| n | 5 | 7 | 5 | 5 |  |
| mean | 1 | 3.206 | 5.168 | 4.457 |  |
| SE | 0.3399 | 1.009 | 1.995 | 1.626 |  |
| ANOVA |  |  |  | F (DFn, DFd) |  |
|  |  |  |  | F (3, 18) = 1,698 | |
| P value |  |  |  | P=0,2032 |  |
| **GluA2 βArr granulares** | | | | | |
|  | **C** | **PTZ** | **WIN** | **WIN+PTZ** |  |
| n | 5 | 7 | 5 | 5 |  |
| mean | 1 | 1.023 | 1.438 | 2.595 |  |
| SE | 0.0971 | 0.2191 | 0.4315 | 1.2950 |  |
| ANOVA |  |  |  | F (DFn, DFd) |  |
|  |  |  |  | F (3, 18) = 1,344 | |
| P value |  |  |  | P=0,2915 |  |
| **CB1 βArr granular** | | | | | |
|  | **C** | **PTZ** | **WIN** | **WIN+PTZ** |  |
| n | 5 | 6 | 4 | 3 |  |
| mean | 1 | 1.215 | 2.037 | 1.78 |  |
| SE | 0.1207 | 0.2011 | 0.6751 | 0.1834 |  |
| ANOVA |  |  |  | F (DFn, DFd) |  |
|  |  |  |  | F (3, 14) = 5,869 | |
| P value |  |  |  | P=0,0082 |  |
| Posthoc | C ♀ vs. WIN + PTZ ♀ | | ** | P=0,0053 |  |
|  | PTZ ♀ vs. WIN + PTZ ♀ | | * | P=0,026 |  |
|  | WIN ♀ vs. WIN + PTZ ♀ | | * | P=0,0439 |  |
| **CAS3 granular** | | | | | |
|  | **C** | **PTZ** | **WIN** | **WIN+PTZ** |  |
| n | 4 | 5 | 3 | 4 |  |
| mean | 1 | 1.565 | 1.604 | 1.979 |  |
| SE | 0.1659 | 0.2171 | 0.2448 | 0.3862 |  |
| ANOVA |  |  |  | F (DFn, DFd) |  |
|  |  |  |  | F (3, 12) = 2,282 | |
| P value |  |  |  | P=0,1312 |  |
| **GluA2 granular** | | | | | |
|  | **C** | **PTZ** | **WIN** | **WIN+PTZ** |  |
| n | 5 | 5 | 4 | 3 |  |
| mean | 1 | 0.6994 | 0.7933 | 1.525 |  |
| SE | 0.105 | 0.0888 | 0.08763 | 0.3386 |  |
| ANOVA |  |  |  | F (DFn, DFd) |  |
|  |  |  |  | F (3, 13) = 5,297 | |
| P value |  |  |  | P=0,0132 |  |
| **βArr granular** | | | | | |
|  | **C** | **PTZ** | **WIN** | **WIN+PTZ** |  |
| n | 4 | 5 | 5 | 4 |  |
| mean | 1 | 1.512 | 1.19 | 1.395 |  |
| SE | 0.286 | 0.208 | 0.3741 | 0.1458 |  |
| ANOVA |  |  |  | F (DFn, DFd) |  |
|  |  |  |  | F (3, 14) = 0,6431 | |
| P value |  |  |  | P=0,6000 |  |
| **CB1 granular** | | | | | |
|  | **C** | **PTZ** | **WIN** | **WIN+PTZ** |  |
| n | 5 | 5 | 4 | 4 |  |
| mean | 1 | 1.151 | 0.6793 | 1.131 |  |
| SE | 0.06168 | 0.1688 | 0.07034 | 0.18 |  |
| ANOVA |  |  |  | F (DFn, DFd) |  |
|  |  |  |  | F (3, 14) = 2,529 | |
| P value |  |  |  | P=0,0994 |  |

| TABLE 6 |  |  |  |  |  |
| --- | --- | --- | --- | --- | --- |
| **Time (sec) spent in each stage of epiletic crisis (increasing severity)** | | | | | |
| **Male ♂** | | | **Female ♀** | | |
| **Grade 1** | | | **Grade 1** | | |
|  | **PTZ** | **WINr+PTZ** |  | **PTZ** | **WINr+PTZ** |
| n | 4 | 4 |  | 6 | 6 |
| mean | 849 | 583 |  | 883.3 | 441.3 |
| SE | 187.3 | 171.7 |  | 97.78 | 99.09 |
| unpaired t-test |  | 0.3355 |  |  | 0.0099 |
|  |  |  |  |  | ** |
| **Grade 3** | | | **Grade 3** | | |
|  | **PTZ** | **WINr+PTZ** |  | **PTZ** | **WINr+PTZ** |
| n | 4 | 4 |  | 7 | 6 |
| mean | 6 | 2 |  | 3.429 | 12.33 |
| SE | 6 | 2 |  | 3.429 | 7.805 |
| unpaired t-test |  | 0.5504 |  |  | 0.294 |
|  |  |  |  |  |  |
| **Grade 4** | | | **Grade 4** | | |
|  | **PTZ** | **WINr+PTZ** |  | **PTZ** | **WINr+PTZ** |
| n | 4 | 4 |  | 7 | 6 |
| mean | 48.5 | 84 |  | 15.43 | 50.67 |
| SE | 37.47 | 44.75 |  | 5.818 | 33.98 |
| unpaired t-test |  | 0.5654 |  |  | 0.2923 |
|  |  |  |  |  |  |
| **Grade 5** | | | **Grade 5** | | |
|  | **PTZ** | **WINr+PTZ** |  | **PTZ** | **WINr+PTZ** |
| n | 4 | 4 |  | 6 | 5 |
| mean | 74 | 172.5 |  | 327 | 70 |
| SE | 38.69 | 93.33 |  | 77 | 44 |
| unpaired t-test |  | 0.3672 |  |  | 0.0231 |
|  |  |  |  |  | * |
| **Grade 6** | | | **Grade 6** | | |
|  | **PTZ** | **WINr+PTZ** |  | **PTZ** | **WINr+PTZ** |
| n | 4 | 4 |  | 7 | 6 |
| mean | 11.5 | 13.5 |  | 30 | 20.67 |
| SE | 4.5 | 5.377 |  | 8.327 | 7.186 |
| unpaired t-test |  | 0.7851 |  |  | 0.4226 |
|  |  |  |  |  |  |
| **Number of times at stage 5 / 0-500 secs** | | | | | |
| **Male ♂** | | | | | |
|  | PTZ | WINr+PTZ |  |  |  |
| n | 4 | 4 |  |  |  |
| mean | 78.5 | 144.5 |  |  |  |
| SE | 45.66 | 69.67 |  |  |  |
| unpaired t-test |  | 0.4583 |  |  |  |
|  |  |  |  |  |  |
| **Number of times at stage 5 / 500-1000 secs** | | | | | |
| **Male ♂** | | | | | |
|  | PTZ | WIN+PTZ |  |  |  |
| n | 4 | 4 |  |  |  |
| mean | 4 | 4 |  |  |  |
| SE | 0 | 32.5 |  |  |  |
| unpaired t-test | 0 | 28.03 |  |  |  |
|  |  | 0.2903 |  |  |  |
| **Number of times at stage 5 / 1000-1500 secs** | | | | | |
| **Male ♂** | | | | | |
|  | PTZ | WIN+PTZ |  |  |  |
| n | 4 | 4 |  |  |  |
| mean | _ | _ |  |  |  |
| SE | _ | _ |  |  |  |
| unpaired t-test |  |  |  |  |  |
|  |  |  |  |  |  |
| **Number of times at stage 5 / 0- 500 secs** | | | | | |
| **Female ♂** | | | | | |
|  | PTZ | WINr+PTZ |  |  |  |
| n | 6 | 6 |  |  |  |
| mean | 169.7 | 42.33 |  |  |  |
| SE | 43.53 | 26.01 |  |  |  |
| unpaired t-test | | 0.0308 |  |  |  |
|  |  | * |  |  |  |
|  |  |  |  |  |  |
| **Number of times at stage 5 / 500-1000 secs** | | | | | |
| **Female ♂** | | | | | |
|  | PTZ | WIN+PTZ |  |  |  |
| n | 6 | 6 |  |  |  |
| mean | 90.67 | 72.33 |  |  |  |
| SE | 49.41 | 56.37 |  |  |  |
| unpaired t-test |  | 0.8117 |  |  |  |
|  |  |  |  |  |  |
| **Number of times at stage 5 / 1000-1500 secs** | | | | | |
| **Female ♂** | | | | | |
|  | PTZ | WIN+PTZ |  |  |  |
| n | 6 | 6 |  |  |  |
| mean | 0 | 41.67 |  |  |  |
| SE | 0 | 40.48 |  |  |  |
| unpaired t-test |  | 0.3276 |  |  |  |

| TABLE 7 |  |  |  |  |  |  |  |
| --- | --- | --- | --- | --- | --- | --- | --- |
| **Latency (sec) to reach each stage of epiletic crisis (increasing severity)** | | | | | |  |  |
| **Male ♂** | | | **Female ♀** | | |  |  |
| **Grade 1** | | | | | |  |  |
|  | **PTZ** | **WINr+PTZ** |  | **PTZ** | **WINr+PTZ** |  |  |
| n | 4 | 4 |  | 7 | 6 |  |  |
| mean | 58 | 59.5 |  | 116.3 | 152.7 |  |  |
| SE | 8.042 | 14.5 |  | 14.37 | 23.52 |  |  |
| unpaired t-test |  | 0.8731 |  |  | 0.1997 |  |  |
|  |  |  |  |  |  |  |  |
| **Grade 4** | | | | | |  |  |
|  | **PTZ** | **WINr+PTZ** |  | **PTZ** | **WINr+PTZ** |  |  |
| n | 3 | 4 |  | 5 | 4 |  |  |
| mean | 153.3 | 110 |  | 231.2 | 168 |  |  |
| SE | 61.43 | 10.68 |  | 66.5 | 16.02 |  |  |
| unpaired t-test |  | 0.4502 |  |  | 0.4371 |  |  |
|  |  |  |  |  |  |  |  |
| **Grade 5** | | | | | |  |  |
|  | **PTZ** | **WINr+PTZ** |  | **PTZ** | **WINr+PTZ** |  |  |
| n | 3 | 4 |  | 6 | 5 |  |  |
| mean | 198.7 | 158 |  | 161.7 | 503.6 |  |  |
| SE | 45.96 | 27.54 |  | 16.58 | 202.7 |  |  |
| unpaired t-test |  | 0.4564 |  |  | 0.0959 |  |  |
|  |  |  |  |  |  |  |  |
| **Grade 6** | | | | | |  |  |
|  | **PTZ** | **WINr+PTZ** |  | **PTZ** | **WINr+PTZ** |  |  |
| n | 3 | 4 |  | 7 | 5 |  |  |
| mean | 180 | 236.5 |  | 206.9 | 402 |  |  |
| SE | 49.27 | 74.38 |  | 56.41 | 206.7 |  |  |
| unpaired t-test |  | 0.5862 |  |  | 0.3139 |  |  |
|  |  |  |  |  |  |  |  |
|  |  |  |  |  |  |  |  |
|  |  |  |  |  |  |  |  |
|  |  |  |  |  |  |  |  |
|  |  |  |  |  |  |  |  |
|  |  |  |  |  |  |  |  |
|  |  |  |  |  |  |  |  |
|  |  |  |  |  |  |  |  |
