# Supplementary material for: Behavioral and Molecular Responses to Exogenous Cannabinoids During Pentylenetetrazol-Induced Convulsions in Male and Female Rats
Source: Front Mol Neurosci. 2022 Aug 9;15:868583. doi: 10.3389/fnmol.2022.868583 (PMC9488559; doi:10.3389/fnmol.2022.868583)
Supplement: Supplementary file 2 [file Data_Sheet_1.docx]

Behavioral and molecular responses to exogenous cannabinoids during pentylenetetrazol - induced convulsions in male and female rats.

Supplementary Methods

**Behavioral evaluation**. Grooming and locomotor activity. Grooming and locomotion activity were measured for 25 minutes, one hour after an injection of DMSO or WIN. Grooming was evaluated as the time spent by the animal performing repetitive paw strokes and body licking as has been established previously (Kalueff et al., 2016). Locomotor activity was measured as the distance travelled and the time spent motionless during 25 min. Both measurements were performed offline using an animal tracking software, ToxTrac (Rodriguez et al., 2018, 2017).

**Injection Protocol**. Additional to the injections described in the “Methods” section of the paper, a small group of animals received an initial i.p. injection of the CB1R antagonist AM251 (“AM251+WIN+PTZ” group) at 1mg/kg 30 min before the WIN injection, to corroborate that the observed results are due to CB1R activation.

**Estrous cycle determination**. To establish the stage of the estrous cycle in females

vaginal smears were analyzed as previously described (Goldman et al., 2007). Only distinct stages (e.g. estrus) were considered, not mixed ones (e.g. estrus/proestrus).

**Western Blots**. To evaluate the success of our cellular fractioning method (see “Methods” on the main paper), we examined the intensity of the membrane bound protein N-cadherin and the cis-Golgi matrix protein GM130 in our “membrane” and “cytoplasmic” partitions. As expected, we observed higher levels of N-cadherin in our membrane fraction compared to the cytoplasm fraction (**Figure 3A, 3B**)(**Suppl. Table 3**). We also obtained higher levels of GM130 in our membrane fraction, which suggests that membrane-bound proteins from internal organelles were also part of our membrane fraction (**Figure 3A, 3C**)(**Suppl. Table 3**). Tubulin was used as the loading control protein, as, despite being a cytoplasmatic protein, it has a strong associations with plasmatic and organelle membranes (Wolff, 2009). Our results indicate our “membrane” fraction was enriched in membrane-bound proteins from the plasmatic membrane but also contained intracellular membranes, though we cannot rule out some contamination from cytoplasmic proteins, or, conversely, of membrane-bound proteins (e.g. from small organelles or vesicles) in the cytoplasmic fraction.
