# Supplementary figures and images for: Behavioral and Molecular Responses to Exogenous Cannabinoids During Pentylenetetrazol-Induced Convulsions in Male and Female Rats
Source: Front Mol Neurosci. 2022 Aug 9;15:868583. doi: 10.3389/fnmol.2022.868583 (PMC9488559; doi:10.3389/fnmol.2022.868583)

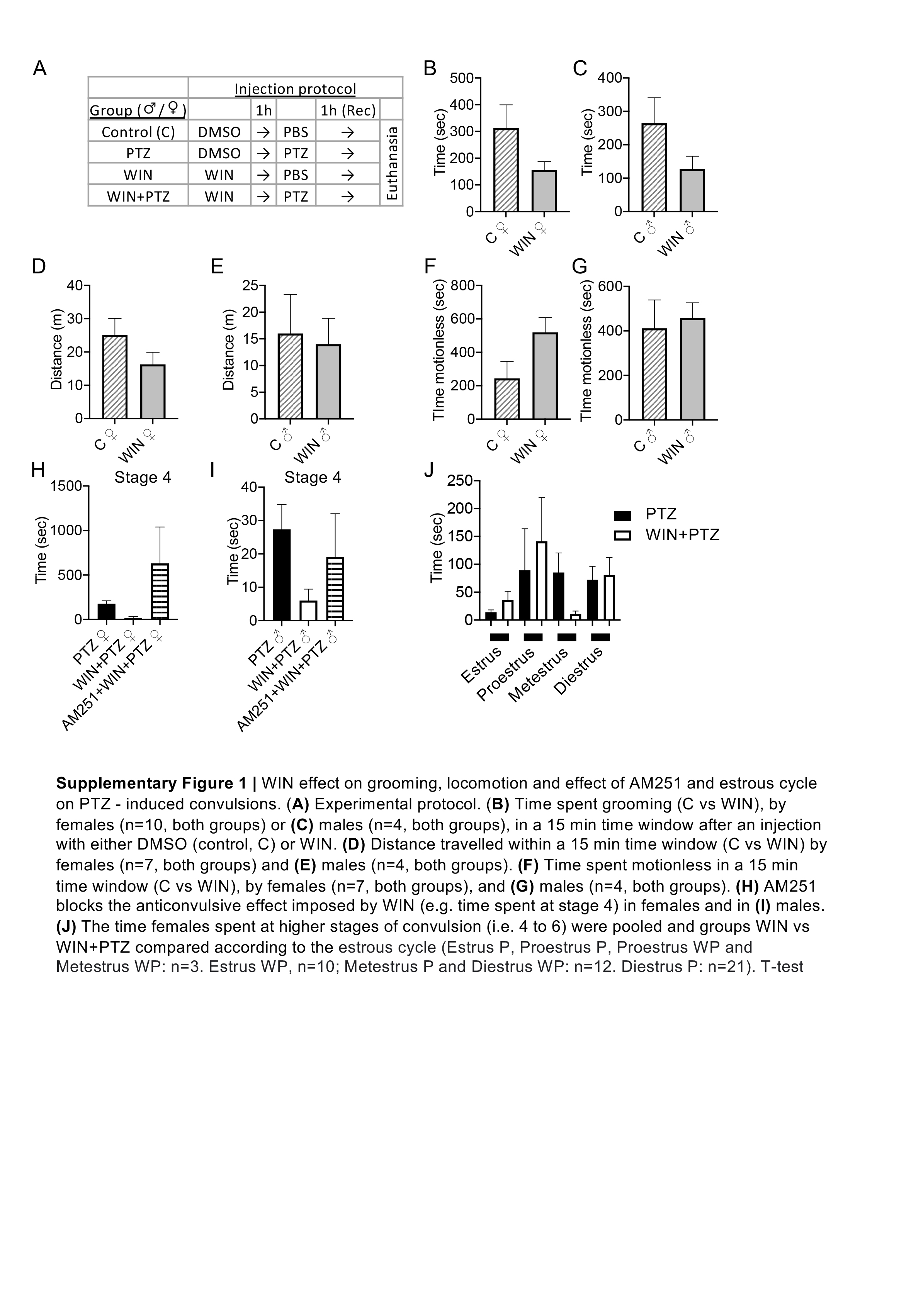

Supplement: Supplementary file 3 [file image_1.jpg]

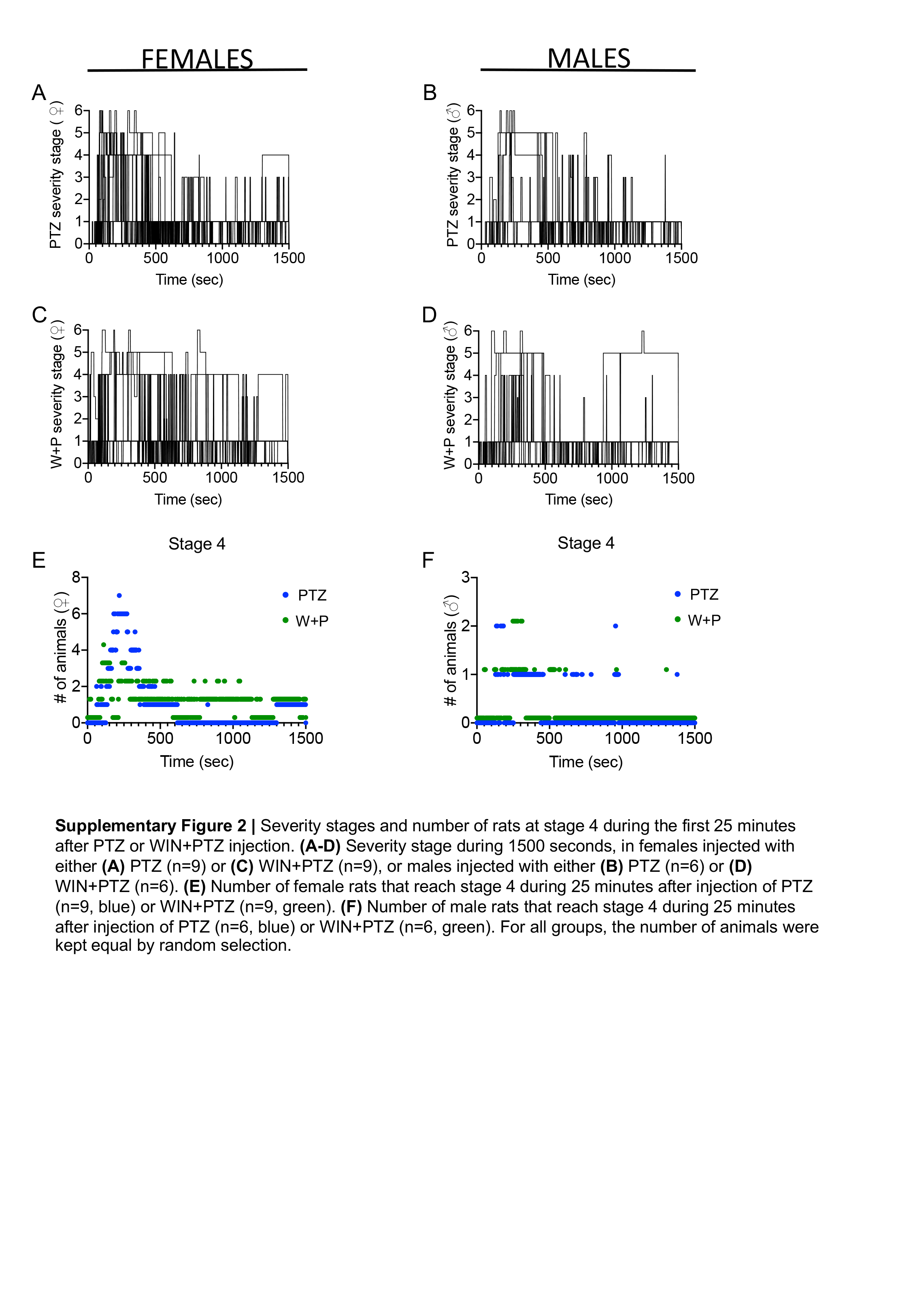

Supplement: Supplementary file 4 [file image_2.jpg]

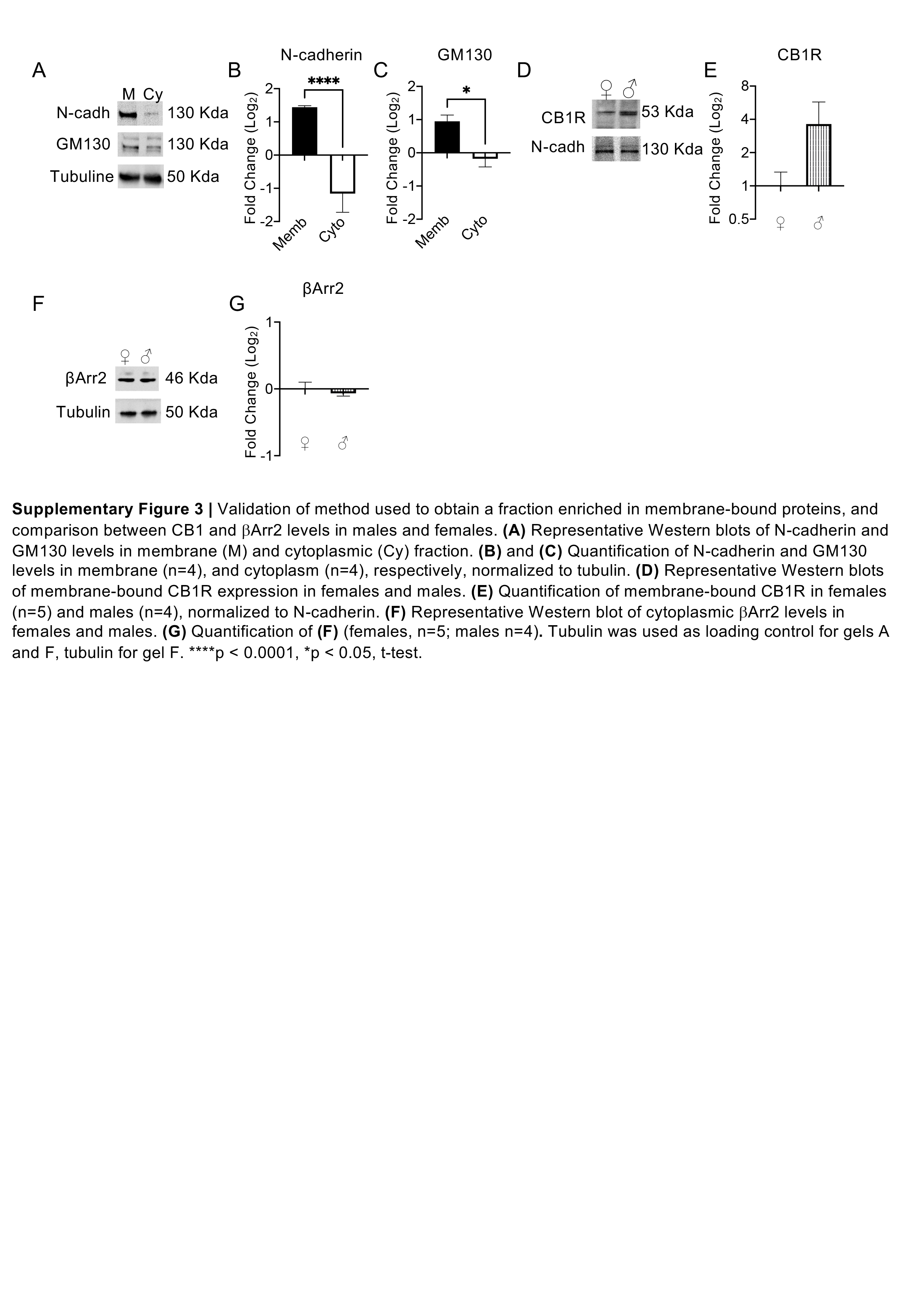

Supplement: Supplementary file 5 [file image_3.jpg]

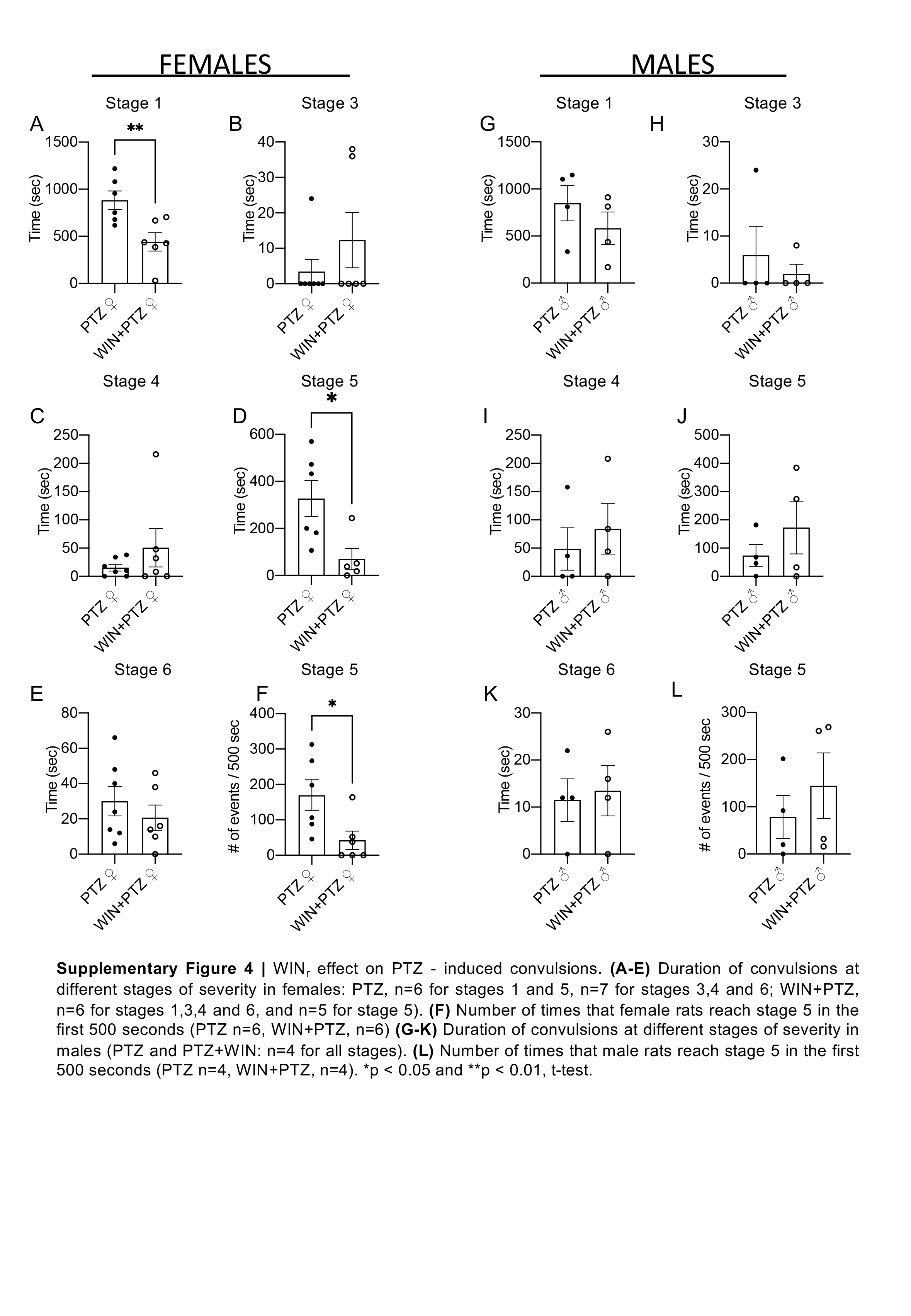

Supplement: Supplementary file 6 [file image_4.jpg]

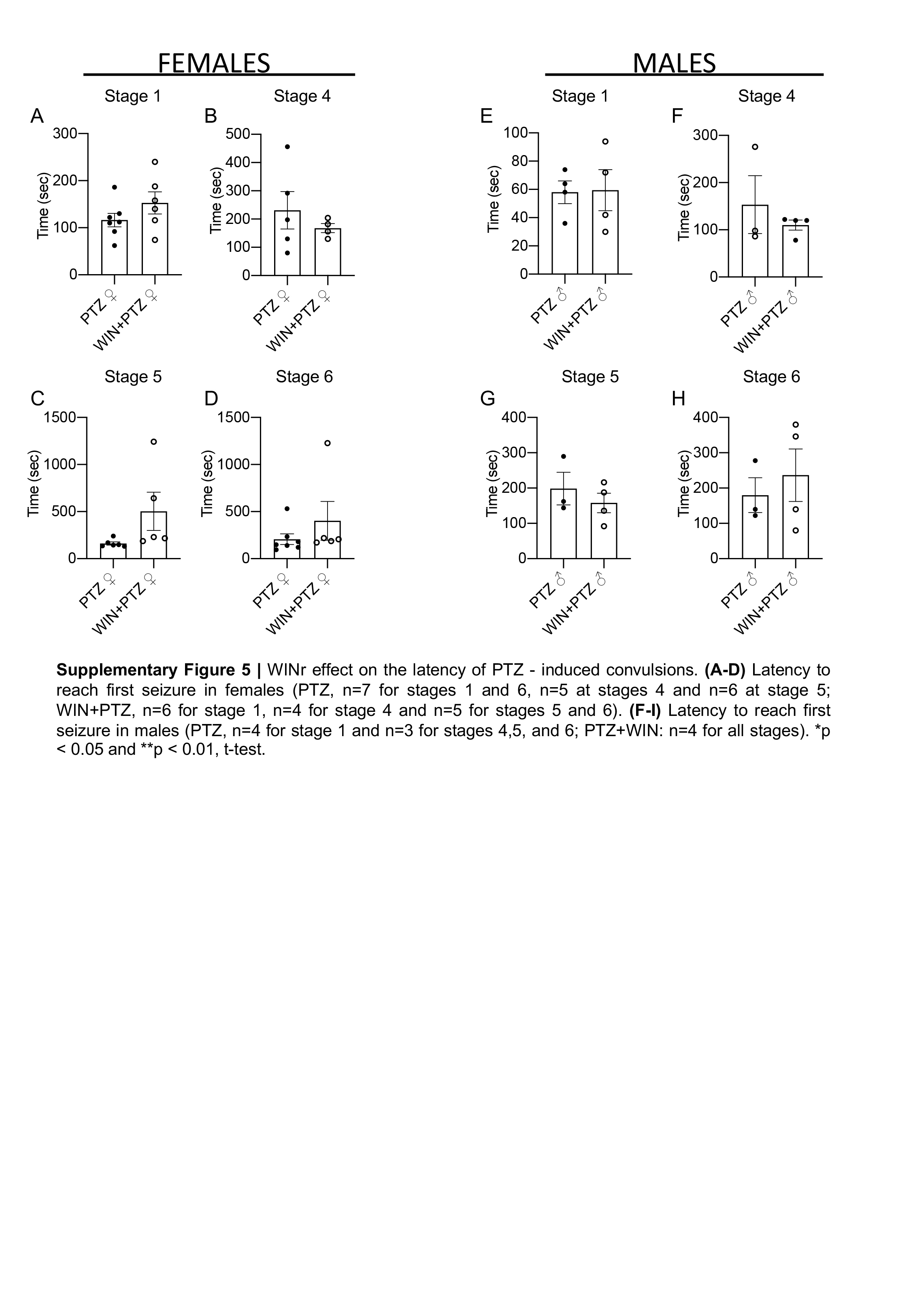

Supplement: Supplementary file 7 [file image_5.jpg]

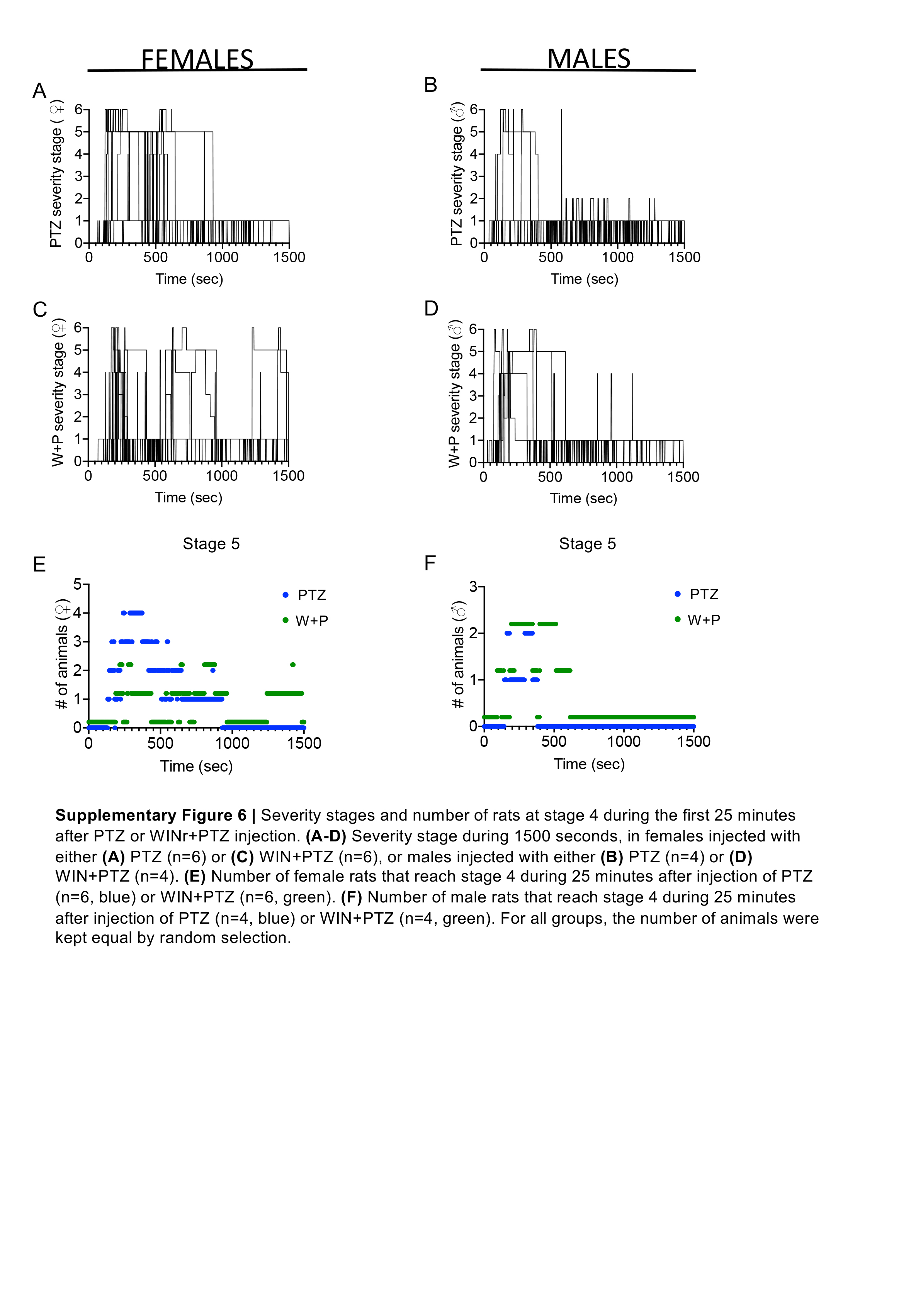

Supplement: Supplementary file 8 [file image_6.jpg]

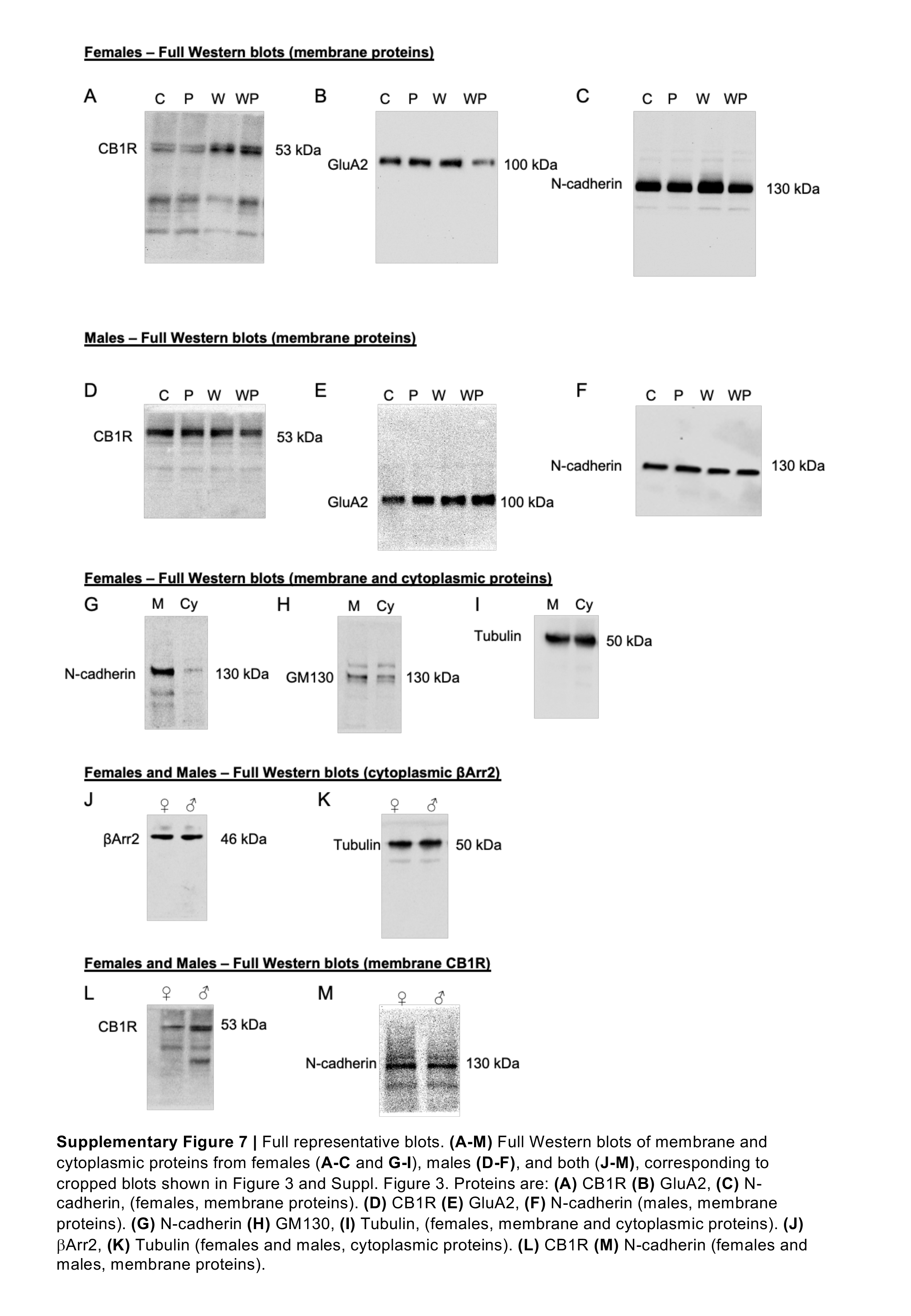

Supplement: Supplementary file 9 [file image_7.jpg]

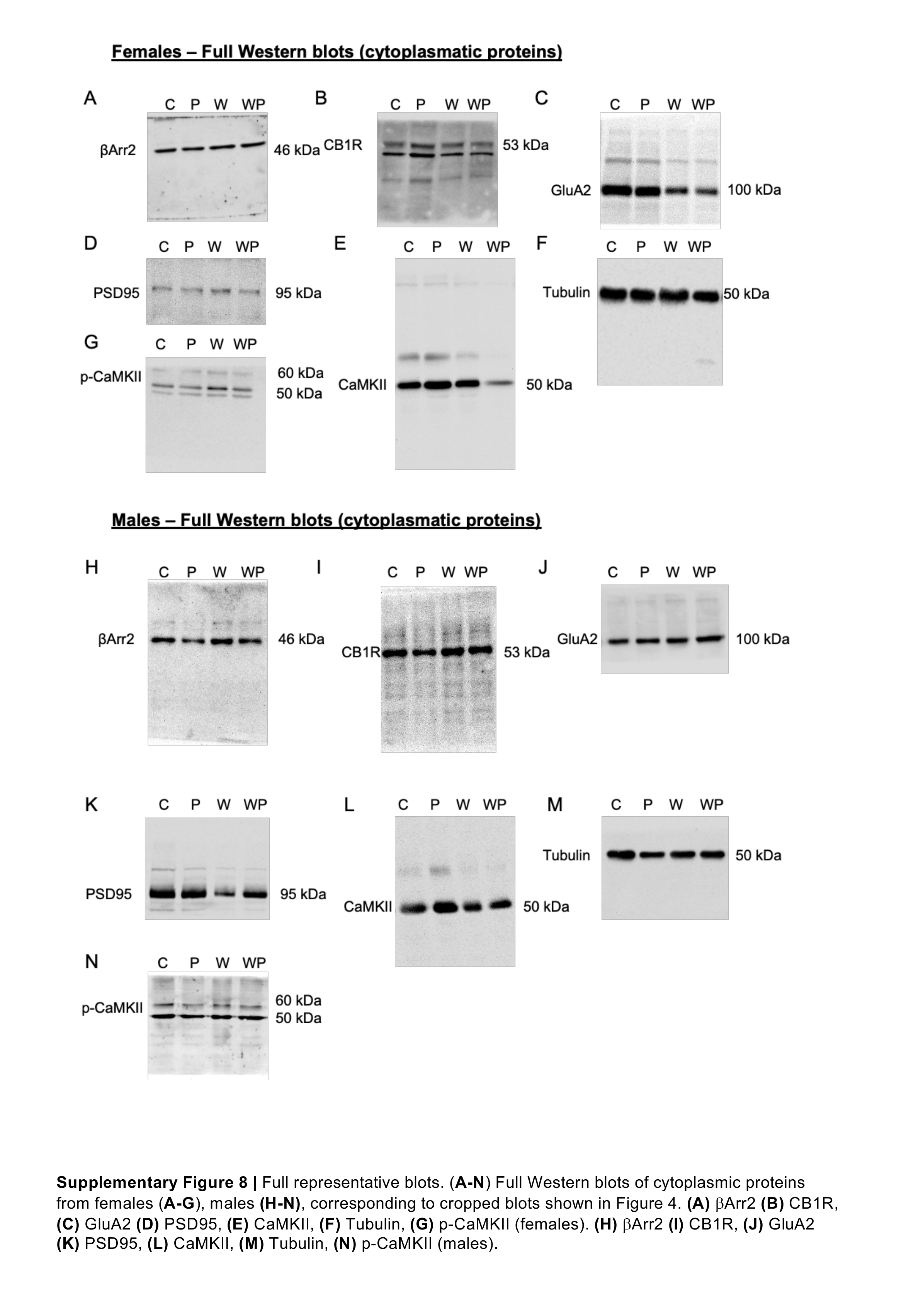

Supplement: Supplementary file 10 [file image_8.jpg]
